# Lactate and lactylation: molecular insights into histone and non-histone lactylation in tumor progression, tumor immune microenvironment, and therapeutic strategies

**DOI:** 10.1186/s40364-025-00849-0

**Published:** 2025-10-24

**Authors:** Shuying Xiao, Suhang Zhang, Kai Sun, Qibo Huang, Qilin Li, Chuanyu Hu

**Affiliations:** 1 Department of Stomatology, Tongji Hospital, Tongji Medical College, Huazhong University of Science and Technology, Wuhan, 430030 China; 2 School of Stomatology, Tongji Medical College, Huazhong University of Science and Technology, Wuhan, 430030 China; 3 Hubei Province Key Laboratory of Oral and Maxillofacial Development and Regeneration, Wuhan, 430022 China; 4 Hepatic Surgery Center, Tongji Hospital, Tongji Medical College, Huazhong University of Science and Technology, Wuhan, 430030 China

**Keywords:** Lactate, Lactylation, Cancer, Immunotherapy

## Abstract

Investigating cancer metabolism is of paramount importance for understanding tumor biology and developing novel therapeutic strategies. Lactylation, a posttranslational modification facilitated by the glycolytic product lactate, plays a crucial role in regulating oncogenic signalling pathways. This review provides a comprehensive analysis of lactate metabolism, including its biosynthesis, compartmentalized transport, enzymatic network and structural features of lactate dehydrogenases, transporters, lactyltransferases and deacetylases. These enzymes contribute to malignant tumor progression through metabolic reprogramming and modulation of the immune microenvironment. Importantly, we emphasize that integrating cancer subtype-specific lactylation profiles with core signatures reveals promising therapeutic opportunities for targeting lactate shuttles, histone, and nonhistone lactylation mechanisms, and transcriptional networks regulated by lactylation. In the present review, we highlight the significant potential of targeting glycolysis and lactylation modifications in tumors to improve the efficacy of cancer treatments.

## Introduction

In 1780, Carl Wilhelm Scheele discovered lactic acid in milk [1]. Since its discovery, lactic acid has often been regarded as a hypoxic metabolic waste product with a variety of harmful effects. Not until the 1980 s did the discovery of the intercellular lactate shuttle mechanism enable a more comprehensive understanding of the role of lactic acid in the whole-body metabolic process [2]. The “circulating turnover flux” of a metabolite refers to the balance between the total flux of the metabolite entering the blood from the tissues and the total consumption of the metabolite by the tissues under a pseudosteady state. Such measurements of lactic acid have been performed several times [3, 4]. In rodents and humans, the circulating turnover flux of lactic acid in the fasting state is approximately twice that of glucose, which means that the two are equivalent in terms of carbon atoms [5]. These findings indicate that in cancer cells, pyruvate often fails to enter the tricarboxylic acid (TCA) cycle and is instead converted to lactate—a shift known as the Warburg effect—and is released into the blood. In addition to the fate of pyruvate, the regulation of glucose uptake via glucose transporters plays a crucial role in controlling glycolytic flux.

Glucose transporters are broadly expressed across tissues, and lactate transporters, while equally widespread, exhibit cell type-specific patterns that enable extensive intertissue lactate shuttling [5, 6]. This expression pattern suggests that lactate may act as an auxiliary energy substrate in various tissues under specific physiological and pathological conditions [7–9]. Furthermore, lactic acid acts as a signalling molecule with autocrine, paracrine, and endocrine effects by activating the cell surface receptors G-protein-coupled receptor 81 (GPR81) and G-protein-coupled receptor 132 (GPR132) [1, 10, 11]. For example, the activation of GPR132 initiates a PKA–AMPK–NAD⁺–SIRT1 signalling cascade that stabilizes the MYC proto-oncogene, a bHLH transcription factor, promotes the proliferation of proresolving macrophages, and mediates tissue repair [12, 13]. Conversely, the GPR81–Gi–Gβγ–RhoA/ROCK1–p38 pathway has been shown to drive tumor-induced cachexia [14]. In the 1920 s, Otto Warburg first observed that tumors consumed more glucose than surrounding normal tissues did; thus, he proposed the phenomenon of aerobic glycolysis. These findings indicate that lactic acid often accumulates in tumors, suggesting that lactic acid may play a role in tumor development. Studies have shown that lactic acid can drive the progression of cancer through multidimensional mechanisms, such as regulating energy metabolism, remodelling the tumor microenvironment, and influencing epigenetic processes [15–20]. Of particular note is the lactate-mediated lactylation modification. Lactylation can be regarded as a landmark discovery in lactate research, as it builds a bridge between epigenetics and metabolic reprogramming [16, 21, 22]. In this review, we summarize the processes through which lactic acid is produced and transported to exert its effects and particularly elaborate on the role of lactylation caused by the accumulation of lactic acid in tumors. The identified lactylation sites commonly found in various tumors are presented in diagrams, and cancer subtype-specific lactylation profiles are established. We have also summarized structural insights into lactate and lactylation-related proteins, providing references for targeted therapy. Finally, we briefly summarize the targeted therapeutic methods for lactylation-related sites.

## The production, transportation, and function of lactate

### Two pathways of lactate production

In cancer, lactate production occurs mainly through two pathways, the glycolysis pathway and the glutamine pathway, which are described below.

#### The glycolytic pathway of lactate production

The glycolysis process is abnormally active in cancer cells, even when oxygen is abundant. Glucose is rapidly taken up and undergoes a series of enzymatic reactions before being ultimately converted into a large amount of lactate. This phenomenon is known as the Warburg effect, which was first discovered and proposed by the German physiologist Otto Warburg in the 1920s. Under aerobic conditions, normal cells generate energy efficiently through aerobic respiration in mitochondria, completely oxidizing glucose into carbon dioxide and water. This process produces a large amount of adenosine triphosphate (ATP). However, cancer cells are different. Even in an aerobic environment, cancer cells tend to preferentially utilize the glycolysis pathway.

During glycolysis, one molecule of glucose is catalysed by a series of enzymes and decomposed into two molecules of pyruvate, while a small amount of ATP and nicotinamide adenine dinucleotide (NADH) are produced. Under normal aerobic conditions, pyruvate will enter the mitochondria and further participate in the tricarboxylic acid cycle to complete thorough oxidation. However, in cancer cells, most pyruvate does not enter the mitochondria. Instead, through the action of lactate dehydrogenase (LDH), it is reduced to lactate by NADH, and at the same time, NADH is reoxidized to NAD⁺ to maintain the continuous progression of glycolysis. Although this metabolic mode produces less ATP than aerobic respiration, the rate of glycolysis is much faster than that of aerobic respiration, which can quickly provide energy for cancer cells to meet their needs for rapid proliferation. Moreover, a large number of intermediate metabolites produced during the glycolysis process can also provide raw materials for the biosynthesis of nucleotides, fatty acids, amino acids, etc., in cancer cells, supporting the rapid growth and division of cancer cells. During this process, MYC and HIF-1 are activated by hypoxia. Both can regulate the activities of hexokinase 2 (HK2) and pyruvate kinase (PK). MYC can also regulate the activity of phosphofructokinase (PFK). These three enzymes are the key enzymes in the glycolysis pathway. MYC and HIF-1 can also activate lactate dehydrogenase A (LDHA) and inhibit lactate dehydrogenase B (LDHB); namely, they promote the conversion of pyruvate to lactate and inhibit the reverse process [4, 23, 24].

#### The glutamine pathway of lactate production

In addition to glucose, cancer cells also rely on the glutamine pathway to produce lactate for energy, maintaining cell growth and proliferation even in the absence of glucose. Glutamine is converted to glutamate by glutaminase, and glutamate is then converted to α-ketoglutarate by glutamate dehydrogenase (GDH) and transaminase. α-Ketoglutarate enters the TCA cycle and, through the malate–pyruvate pathway, is eventually converted to lactate by LDHA [25, 26]. Studies have shown that inducing MYC expression can lead to the induction of the expression of glutamine transporters, glutaminase, and lactate dehydrogenase A [27]. Lactate accumulation can, in turn, promote glutamine decomposition, forming a positive feedback loop for lactate formation [28].

### Lactate transport proteins

#### The lactate transporters monocarboxylate transporters (MCTs)

Lactate transport is mediated primarily by MCTs. MCTs, encoded by the solute carrier 16 (SLC16) gene family, are responsible for the transport of monocarboxylate molecules (such as lactate and pyruvate) into and out of cells. To date, 14 MCTs have been identified, but only MCT1/SLC16A1, MCT2/SLC16A7, MCT3/SLC16A8, and MCT4/SLC16A3 (hereinafter referred to as MCTs) transport monocarboxylate ions along with protons [29]. Among these proteins, MCT1, MCT2, and MCT4 play important roles in tumor progression [30]. The uneven distribution of oxygen in tumors creates relatively oxygen-rich and hypoxic regions. Oxygen-rich cancer cells can undergo oxidative phosphorylation, while hypoxic cancer cells can undergo glycolysis. MCT4 is a low-affinity lactate transporter expressed in glycolytic cells that promotes lactate efflux [31]. In contrast, MCT1 has a high affinity for lactate and is expressed primarily in cells undergoing oxidative phosphorylation, promoting lactate influx and oxidative energy production. This process represents a form of metabolic symbiosis in tumors known as the lactate shuttle [4].

#### Regulation of MCT expression

MCT expression is influenced by various factors. Hypoxia can directly induce the expression of the MCT1, MCT2, and MCT4 genes by activating HIF-1 [32, 33]. MYC signalling can directly trigger MCT1 and MCT2 expression [34, 35]. Wnt activation can promote MCT1 expression [36]. Nuclear factor-kappa B (NF-κB) signalling and the loss of tumor protein p53 (p53) function can further trigger MCT1 transcription [34]. Colon cancer 1 (MACC1) signalling has been reported to induce MCT1 transcription in gastric cancer cell lines [37]. PGC-1α–oestrogen-related receptor α (ERRα) signalling supports MCT1/SLC16A1 transcription [38]. Glutamine increases HIF-1 activity and promotes MCT4 expression [39]. Extracellular lactate acting on GPR81 can trigger a signalling cascade that increases the expression of the MCT1 and MCT4 genes in pancreatic ductal adenocarcinoma (PDAC) cell lines [40]. These factors promote MCT expression, but reduced MCT expression may be due to hypermethylation of gene promoters, leading to MCT1 silencing in breast cancer [41] and MCT4 silencing in colorectal cancer [29, 42].

### The function of lactate

#### Lactate as a fuel for cellular metabolism

Lactate serves as an energy source through the lactate shuttle between cancer cells, facilitating the self-sufficiency and sustainability of cancer cells [43]. A form of metabolic symbiosis between tumor cells and cancer-associated fibroblasts known as the reverse Warburg effect has also been identified. In this process, the aerobic glycolysis of nontumor cells, such as cancer-associated fibroblasts, is increased, and a large amount of glucose is converted into metabolites such as lactate. On the other hand, tumor cells exhibit relatively weaker glycolysis and rely more on obtaining metabolites such as lactate from external cells such as fibroblasts. They then generate large amounts of ATP through the aerobic mitochondrial metabolic pathway to meet the high energy demands of activities such as tumor cell proliferation, invasion, and metastasis. In this model, cancer cells are thought to create a “pseudohypoxic” environment for cancer-associated fibroblasts by secreting hydrogen peroxide, which activates HIF-1α and MCT4 expression in stromal cells, promoting glycolysis. The newly generated lactate is then exported from stromal cells by MCT4 and imported into cancer cells by MCT1 [44, 45]. In human non-small cell lung cancers (NSCLCs), Faubert B et al. discovered that compared with glucose, lactate plays a dominant role in the TCA cycle [46]. In the tumor microenvironment, fibroblasts can utilize extracellular lactic acid to maintain the TCA cycle [47].

#### Lactate as a signalling molecule

**Fig. 1:**
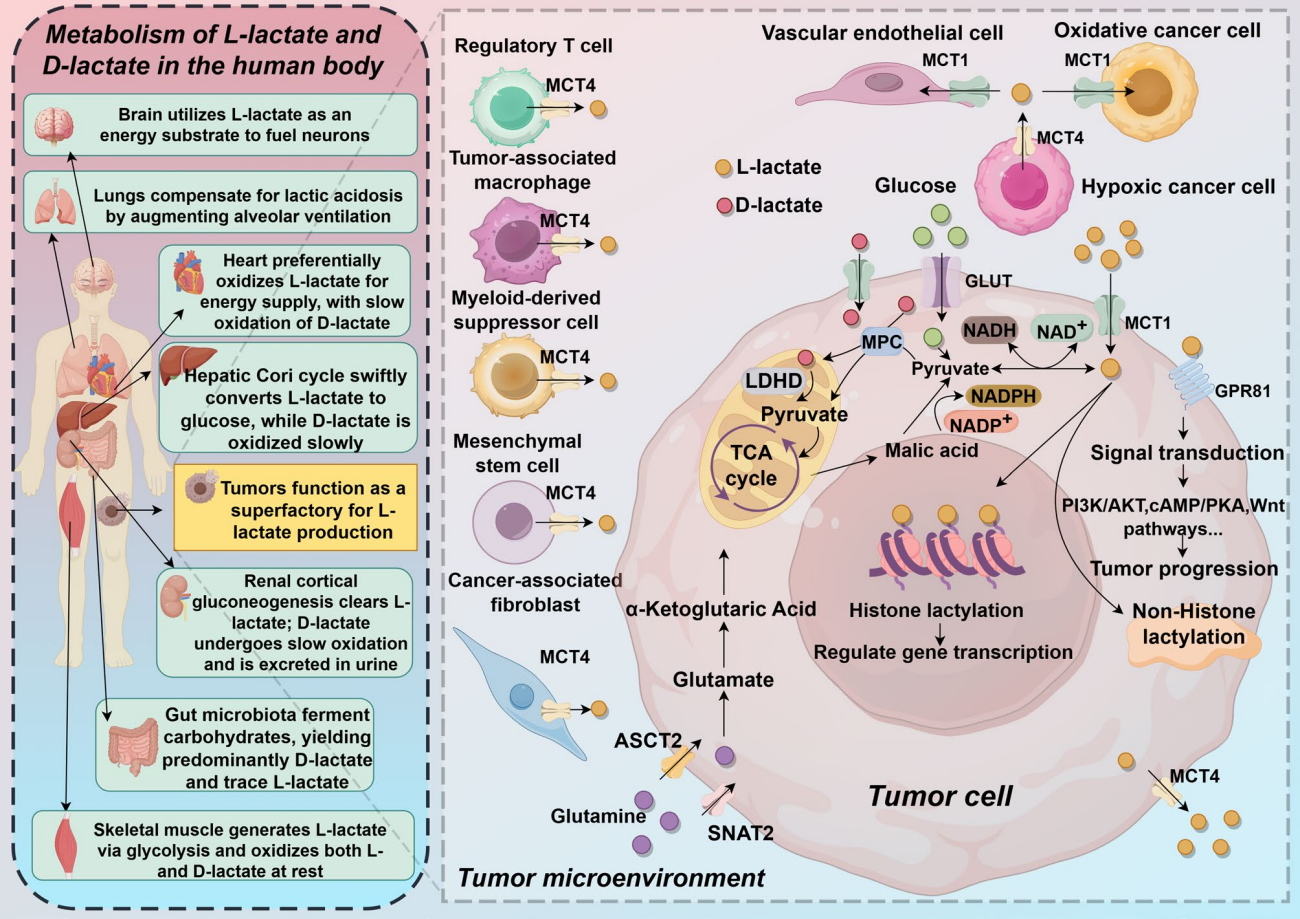
The production, transportation, and function of lactate

As a signalling molecule, lactate acts on GPR81 to regulate downstream signalling pathways and can also enter cells for regulation [48]. Lactate can promote cancer metastasis, as evidenced by its role in promoting the epithelial–mesenchymal transition (EMT) and angiogenesis in tumors [49]. The epithelial‒mesenchymal transition is a reversible cellular program in which epithelial cells gradually lose their functional and morphological characteristics and acquire a mesenchymal cell morphology. Lactate primarily promotes the EMT through the transforming growth factor-β (TGF-β) pathway [50]. The lactate shuttle between tumor cells and vascular endothelial cells aids in vascular formation. The specific mechanism involves the entry of lactate into cells via MCT1, where it acts as a signalling molecule to stabilize HIF-1α and activate the autocrine NF-κB/IL-8 (CXCL8) pathway, promoting tumor cell migration and angiogenesis [49] (Fig. 1).

#### Lactate regulates immunity

Tumor-associated macrophages (TAMs) can be classified into classically activated (M1) or activated (M2) groups [51]. The M2 polarization if TAMs can regulate vascular endothelial growth factor (VEGF) production, aiding in tumor angiogenesis, invasion, and immune suppression [52]. Lactate inhibits the M1 polarization of TAMs, inducing their differentiation into the M2-like phenotype. The specific mechanism involves the induction of increased intracellular reactive oxygen species (ROS) levels by lactate, promoting nuclear factor-erythroid 2-related factor 2 (Nrf2) nuclear accumulation, and leading to M2 polarization. Nrf2 can upregulate the expression of vascular endothelial growth factor (VEGF) and arginase-1 (ARG1), and the stabilization of HIF-1α also promotes M2 polarization [53, 54]. Additionally, studies have shown that lactate promotes the ERK/signal transducer and activator of transcription 3 (STAT3) pathway, inducing downstream phenotypic transformation [55, 56]. Lactate can also act on GPR132 to promote phenotypic transformation [57]. G-protein-coupled receptor 65 (GPR65) is selectively overexpressed on tumor-associated macrophages (TAMs) in gliomas. It senses lactate stimulation through the cAMP/PKA/CREB signalling pathway and promotes high mobility group box 1 (HMGB1) secretion, thereby contributing to tumor progression [58].

Neutrophils play crucial roles in defending against infections as well as activating and regulating innate and adaptive immunity. In cancer, tumor-associated neutrophils (TANs) can play dual roles; for example, immature human CD10⁻CD66b⁺ neutrophils promote T-cell activation, whereas CD10⁺ neutrophils have the opposite effect [59]. Tumor-derived lactate induces programmed death-ligand 1 (PD-L1) expression on neutrophils through the MCT1/NF-κB/cyclooxygenase-2 (COX-2) pathway, thereby inhibiting T-cell cytotoxicity [60]. In brain tumors, hypoxia increases glucose metabolism in CD71⁺ neutrophils, resulting in lactate accumulation and subsequent histone lactylation. This process then regulates the expression of arginase-1, thereby inhibiting T-cell function [61].

The consequences of eosinophil involvement in the tumor microenvironment (TME) remain elusive; however, according to current research, eosinophil infiltration is associated with a favourable tumor prognosis in most cases [62]. Tumor-derived lactate can suppress the function of Group 2 innate lymphoid cells (ILC2s) and reduce eosinophil accumulation, thereby weakening the inhibitory effect on tumor growth [63].

With respect to cytotoxic T and NK cells, studies have shown that lactate in the TME interferes with intracellular signalling and the regulation of cellular function, hindering lactate export from T cells and limiting T-cell proliferation [64, 65]. Tumor-derived lactate also alters the TCA cycle balance, impairing the cytolytic activity of CD8^+^ T cells [66]. In addition to its direct effects, lactate can inhibit the cytotoxic activity of T and NK cells through its acidifying effect or by regulating downstream signalling. Lactate acidosis in the TME selectively targets the downstream signalling pathways of the mitogen-activated protein kinases (MAPKs) p38 and c-Jun n-terminal kinase (JNK)/c-Jun, hindering CTL function, interfering with mammalian target of rapamycin (mTOR) signalling, and leading to impaired NKT cell function [67, 68]. Lactate-induced intracellular acidification inhibits nuclear factor of activated T cells (NFAT), resulting in reduced IFN-γ production [69]. Lactate induces T-cell apoptosis by inactivating NF-κB and reducing NAD^+^ levels [70]. Lactate can also inhibit or increase the expression of ligands or receptors on immune or tumor cells, disrupting the recognition and cytotoxic functions of T and NK cells. Lactate can inhibit the expression of the activating receptor NKp46 on NK cells, weakening their cytotoxic activity [71]. In human lung cancer cells, lactate–GPR81 signalling upregulates PD-L1 expression, interfering with immune cell recognition and killing of tumor cells [72]. Furthermore, lactate can promote SIRT1-mediated deacetylation or degradation of the T-box transcription factor expressed in T cells (T-bet transcription factor), regulating CD4^+^ T-cell polarization and reducing the percentage of helper T-cell 1 (Th1) cells [73]. Lactate signalling in CD4^+^ T cells promotes Th17 cell differentiation and increases interleukin (IL)−17 expression while inhibiting T-cell migration and transport [74]. Lactate stimulation induces the production of IL-6 by cancer-associated fibroblasts, which significantly reduces the expression of Granzyme B (GraB) and IFN-γ in CD8⁺ T cells, impairing their cytotoxic function [75].

However, lactate can inhibit the activity of histone deacetylases, leading to increased acetylation at the H3K27 site of the Tcf7 superenhancer. This modification results in upregulated Tcf7 gene expression and an increased proportion of stem-like TCF-1-expressing CD8⁺ T cells among intratumoral CD3⁺ cells, thereby inhibiting tumor growth. These findings highlight the complexity of the role of lactate in the TME [76].

Dendritic cells (DCs) play an antigen-presenting role in antitumor immunity. In lung cancer, lactate affects the adaptive function of DCs, accelerating antigen degradation and hindering cross-presentation [77]. High concentrations of lactic acid inhibit the differentiation of CD1a^+^CD14^−^ monocyte-derived dendritic cells (MoDCs), promote the production of interleukin-10 (IL-10), and suppress the differentiation of DCs that produce CD1a^+^ interleukin-12 (IL-12), thus leading to immunosuppression [49, 78]. Lactate inhibits the expression of IFN-α and IFN-β in plasmacytoid dendritic cells (pDCs), weakening the antitumor immune response [79]. Lactate promotes the development of CD63⁺ mregDCs by activating SREBP2-dependent signalling. CD63⁺ mregDCs can inhibit antigen cross-presentation by neighbouring DCs through the production of soluble mediators, thereby effectively impairing local CD8⁺ T-cell activation. Additionally, they can support the differentiation of FoxP3⁺ Tregs, leading to the generation of immunotolerant T-cell responses [80]. Lactate can affect cross-presentation by downregulating the expression of membrane transport proteins such as soluble N-ethylmaleimide-sensitive factor attachment protein receptors (SNAREs) and vesicle-associated membrane protein 3 (VAMP3). Moreover, it accelerates antigen degradation in DCs, thereby inhibiting antitumor responses [11].

Tregs participate in tumor immune tolerance by inhibiting the proliferation and stimulation of immune cells while releasing anti-inflammatory cytokines. In a low-glucose, lactate-rich environment, Tregs can take up lactate, and their immunosuppressive function is enhanced. Mechanistically, lactate may promote Treg cell maturation and differentiation by mediating MOESIN K74 lactylation and amplifying TGF-β signalling [81]. Additionally, high expression of forkhead box P3 (FOXP3) in the TME inhibits c-MYC expression and glycolysis, enhances oxidative phosphorylation (OXPHOS), increases NAD^+^ oxidation, and reprograms Treg metabolism to increase the adaptability of Treg cells in a low-glucose, high-lactate TME [82]. Moreover, in a highly glycolytic TME, Tregs actively absorb lactate through MCT1, promoting the translocation of NFAT1 to the nucleus and thereby increasing the expression of the immunosuppressive molecule PD-1 [83]. Elevated intracellular lactate levels promote the activation of XBP1 and upregulate α−1,3-mannosylglycoprotein 2-β-N-acetylglucosaminyltransferase (MGAT1), thereby enhancing oxidative phosphorylation and immunosuppressive function in naive regulatory T cells (Tregₙ) [84]. Lactate induces Foxp3 expression, which in turn promotes ubiquitin-specific peptidase 39 (USP39)-dependent CTLA-4 expression in Tregs, helping them maintain their phenotype and functional status [85].

Myeloid-derived suppressor cells (MDSCs) contribute to immune suppression, including T-cell suppression and innate immune regulation. Tumor-derived lactate can promote MDSC proliferation by increasing the expression of granulocyte‒macrophage colony-stimulating factor (GM-CSF)/IL-6, which inhibits NK cell cytotoxicity [71]. Lactate promotes MDSC differentiation through the Notch/Hes1/MCT2/c-Jun axis [86]. In pancreatic cancer, lactate activates MDSCs through the GPR81/mTOR/HIF-1α/STAT3 pathway, enhancing the immunosuppressive function of MDSCs [4, 87, 88]. ZDHHC9-mediated palmitoylation of LDHA promotes lactate production, leading to increased infiltration of monocytic MDSCs and impairment of the antitumor immune response of effector T cells [89]. Activation of the lactate receptor HCAR1 signalling pathway induces the expression of the chemokines CCL2 and CCL7 in colorectal tumor cells, leading to the recruitment of immunosuppressive CCR2⁺ polymorphonuclear myeloid-derived suppressor cells (PMN-MDSCs) into the tumor microenvironment, thereby resulting in immunosuppression [90] (Table 1).

**Table 1:** Lactate and lactylation in immunity

Categories	Mechanism	Effect	References
TAMs	lactate → ROS↑ → Nrf2 nuclear accumulation → VEGF, ARG1↑ → macrophage M2 polarization	immune suppression	[53, 54]
	lactate → ERK/STAT3 pathway → macrophage M2 polarization	immune suppression	[55]
	lactate → GPR132 → HIF1α, Arg-1 → VEGF → macrophage M2 polarization	immune suppression	[57]
	lactate → TAMs → GPR65 → cAMP/PKA/CREB signal way → HMGB1	tumor progression	[58]
	lactate → H3K18la → VCAM1 → CXCL1↑ → macrophage M2 polarization	immune suppression	[91]
	lactate → H3K18la → RARγ↓ → TRAF6-IL-6-STAT3	tumor growth	[92]
	lactate → RIG-1la → macrophage M2 polarization	immune suppression	[93]
	lactate → MYB↑ → H3K18la → macrophage M2 polarization	immune suppression	[94]
	lactate → H3K18la → TNFSF9↑ → macrophage M2 polarization	immune suppression	[95]
	lactate → H3K18la → GPD2↑ → macrophage M2 polarization	immune suppression	[96]
TAN	lactate → MCT1/NF-κB/COX-2 → TAN → PD-L1↑	impair cytotoxicity	[60]
	hypoxia → TAN → lactate → Arg-1↑	impair cytotoxicity	[61]
Eos	lactate → inhibit ILC2 function → EOS aggregation↓	immune suppression	[63]
T cells	lactate in TME → interfere with intracellular signal transduction	restrict T cell proliferation	[64, 65]
	lactate → affect the TCA cycle	impair cytotoxicity	[66]
	lactate → inhibition of MAPKs p38 and JNK/c-Jun	impair cytotoxicity	[67]
	lactate → intracellular acidification → inhibit the action of NFAT → IFN-γ↓	impair cytotoxicity	[69]
	lactate → NF-κB, NAD^+^↓	T cell apoptosis	[70]
	lactate → GPR81 → PD-L1↑ → IFN-γ↓	impair cytotoxicity	[72]
	lactate → SIRT1 → deacetylation of the T—bet transcription factor → Th1 cells↓	immune suppression	[73]
	lactate → Th17 cells↑ → IL-17↑ → CID	tumor growth	[74]
	lactate → CAFs → IL-6 → GraB, IFN-γ↓ →	impair cytotoxicity	[75]
	lactate → HDAC↓ → Tcf7 H3K27ac↑ → Tcf7, CD8^+^T cells↑	inhibit tumor growth	[76]
NK cells	lactate → inhibition of the mTOR signal pathway	impair cytotoxicity	[68]
	lactate → NKp46↓	impair cytotoxicity	[71]
DC	lactate → inhibit the differentiation of CD1a^+^CD14^−^ MoDCs → IL-12↓,IL-10↑	immune suppression	[49, 78]
	lactate → IFN-α, IFN-β↓	immune suppression	[79]
	lactate → SREBP2 → CD63^+^mregDC → inhibit DCs and promote Treg differentiation	immune suppression	[80]
	lactate → SNARE, VAMP3↓ → inhibit cross-presentation and promote antigen degradation	tumor progression	[11]
Treg cells	lactate → MOESIN K72la → TGF-β → SMAD3 signal pathway → promote the proliferation and differentiation of Treg cells	immune suppression	[81]
	lactate → nuclear translocation of NFAT1 → PD-1↑	immune suppression	[83]
	lactate → APOC2 K70la → Tregs↑	immune suppression	[97]
	lactate → X-box binding protein 1 (XBP1) → MGAT1↑ → TregN oxidative phosphorylation	immune suppression	[84]
	lactate → FOXP3 → Tregs → CTLA-4↑ → maintain Treg function	immune suppression	[85]
MDSC	lactate → GM-CSF/IL-6 → proliferation of MDSCs	immune suppression	[71]
	lactate → Notch/Hes1/MCT2/c-Jun → differentiation of MDSCs	tumor growth	[86]
	lactate → GPR81/mTOR/HIF-1α/STAT3 → MDSC	immune suppression	[4, 87, 88]
	ZDHHC9 → LDHA C163Pa → lactate↑ → MDSC↑	immune suppression	[89]
	lactate → HCAR1 signal pathway → CCL2, CCL7↑ → PMN-MDSCs recruitment	immune suppression	[90]

The figure illustrates the production, transport, and roles of D-lactate and L-lactate.

L-lactate is generated primarily through the glycolysis pathway and the glutamine pathway, whereas D-lactate is produced mainly via non-lactate dehydrogenase routes. Both stereoisomers are transported across cell membranes by monocarboxylate transporters (MCT1, MCT4). The brain, heart, and skeletal muscle preferentially oxidize L-lactate as an energy source. The liver rapidly reconverts L-lactate to glucose via the Cori cycle, while renal cortical gluconeogenesis clears circulating L-lactate. Hypoxic tumors act as “super-factories,” exporting abundant L-lactate through MCT4, acidifying the tumor microenvironment and promoting tumor progression via PI3K/AKT, cAMP/PKA, and Wnt signaling pathways. L-lactate also serves as an epigenetic substrate, regulating gene transcription through histone and non-histone lactylation. Regulatory T cells, tumor-associated macrophages, cancer-associated fibroblasts, and myeloid-derived suppressor cells all respond to extracellular L-lactate, establishing an immunosuppressive milieu. In contrast, gut microbiota fermentation predominantly yields D-lactate (with trace L-lactate). D-lactate is slowly oxidized by the liver and kidneys, and excess D-lactate is excreted in urine. At rest, skeletal muscle and heart can oxidize both lactate stereoisomers, although the capacity for D-lactate oxidation is limited. The lungs compensate for lactic acidosis by increasing alveolar ventilation. Collectively, the differential metabolism of L- and D-lactate orchestrates systemic energy homeostasis, acid–base balance, immune regulation, and tumor biology.

### Introduction to and related functions of D-lactate

Lactic acid is a chiral compound with two optical isomers: D-lactic acid and L-lactic acid [98]. Usually, the lactic acid we refer to is L-lactic acid. Only trace amounts of D-lactic acid are produced in human tissues, but it can accumulate in individuals with certain metabolic disorders [99]. In humans and eukaryotes, the production of D-lactate primarily occurs through non-LDH pathways under abnormal metabolic states, among which the methylglyoxal pathway is the main route. This process involves the conversion of glucose to methylglyoxal, which is then further converted to D-lactate [100]. In addition, D-lactate can also be produced by intestinal microorganisms (such as Lactobacillus). These microorganisms break down glucose to generate D-lactate, which is then absorbed through the intestinal tract and enters the bloodstream [101, 102]. Its metabolic role in healthy tissues remains limited and distinct from that of L-lactate [103]. Although both are chiral molecules with minimal structural differences, they differ in their physiological functions and metabolic pathways. Han et al. found that treatment with D-lactic acid downregulated the expression of M2-related genes (such as ARG1, Fizz, and IL-10) in M2 macrophages in hepatocellular carcinoma, whereas M1-related genes (such as tumor necrosis factor-α (TNF-α), Nos, and IL-12) were upregulated under the same conditions. The morphological structure of M2 macrophages became similar to that of M1 macrophages. Han et al. further proposed that D-lactic acid induces macrophage phenotype switching by inhibiting the phosphatidylinositol 3-kinase (PI3K)/protein kinase (BAKT) pathway in hepatocellular carcinoma models [104–106]. Lv et al. reported that the accumulated D-lactic acid can induce iron-dependent death in tumor cells through the cyclin-dependent kinase 7 (CDK7)–yes-associated protein (YAP)–LDHD axis. This process involves an increase in ROS levels and the intracellular iron content, and at the same time, it reduces the levels of matrix metalloproteinases (MMPs) and glutathione (GSH) in ESCC [107]. Lu et al. found that synthetic D-lactate dimers can inhibit the proliferation and survival of melanoma and squamous cell carcinoma cells, indicating promising prospects for cancer therapy [108]. Cai et al. discovered that D-lactic acid selectively induces mitochondrial oxidative phosphorylation, reversibly inhibits aerobic glycolysis in cancer cell lines and proliferating primary cells in an ATP-dependent manner, and enables cells to grow on respiration-dependent bioenergetic substrates. In primary T cells, D-lactic acid increases cell proliferation and augments effector functions [109]. However, some studies have reported that D-lactate, similar to L-lactate, can enhance DNA repair and affect the resistance of cervical cancer cells to anticancer drugs by inhibiting histone deacetylases and activating HCAR1 [110]. In summary, D-lactate plays a crucial role in regulating cancer cell ferroptosis and macrophage activity, but its exact function in cancer metabolism remains to be investigated.

D-Lactate can participate in protein lactylation through the nonenzymatic covalent modification (NECM) pathway. Lactoylglutathione (LGSH) generated by the glyoxalase (GLO) pathway can directly serve as a lactyl donor, transferring the D-lactyl group to the lysine residues of target proteins to form D-lactyl modification (K(D-la)) [111]. D-Lactate-mediated D-lactylation of the K310 site of RelA blocks NF-κB transcriptional activity and reduces the expression of inflammatory factors (such as IL-6 and TNF-α) [112].

## Lactylation-related enzymes and lactylation in cancer

Lactylation was first reported by Zhang et al. in 2019, who identified 28 lactylation sites on core histones in human and mouse cells [113]. Since its discovery, lactylation has been widely reported in different parts of organisms and on various proteins and is not limited to histones.

### The enzyme that synthesizes the lactyl group donor lactyl-CoA

Lactyl-CoA often serves as the primary donor for lactyl groups. Liu et al. found that lactate accumulation due to the Warburg effect maintains the function of the lactyl-CoA synthetase of GTP-specific succinyl-CoA synthetase (GTPSCS), promoting histone lactylation [114]. Zhu et al. showed that epidermal growth factor receptor (EGFR) activates extracellular signal-regulated kinase (ERK), promoting the ERK-mediated phosphorylation of S267 of acetyl-CoA synthetase 2 (ACSS2) and the nuclear translocation of acyl-CoA synthetase short chain family member 2 (ACSS2). ACSS2 subsequently forms a complex with lysine acetyltransferase 2 A (KAT2A), facilitating histone lactylation. In this process, ACSS2 functions as a genuine lactyl-CoA synthetase and converts lactate into lactyl-CoA [115].

### Writers, erasers, and readers of lactylation

Lactylation requires “writers”, enzymes that add or introduce lactyl groups to substrate molecules. The most extensively studied lactyltransferase is E1A binding protein p300 (p300). In pancreatic ductal adenocarcinoma (PDAC), p300 promotes H3K18la, and H3K18la stimulates threonine tyrosine kinase (TTK) and BUB1 mitotic checkpoint serine/threonine kinase B (BUB1B) transcription, promoting PDAC cell cycle progression, tumor progression, and glycolysis, which in turn increase H3K18la levels, leading to the formation of a feedback loop [116]. In intrahepatic cholangiocarcinoma (iCCA), due to active glycolysis, nucleolin (NCL) is lactylated at lysine 477 by the acyltransferase p300, promoting iCCA cell proliferation and invasion [117]. Another study revealed that nucleus-localized GTPSCS interacts with p300 to coregulate H3K18la and GDF15 expression and promote glioma proliferation and radioresistance [114]. As a homologue of p300, CREB-binding protein (CBP) also exhibits lactylation activity. After DNA damage occurs, the interaction between MRE11 and CBP increases, and MRE11 is specifically lactylated at K673 in its second DBD, enhancing its DNA-binding ability and promoting end resection and HR repair [118]. Studies have shown that knocking out general control nonrepressed 5 (GCN5) significantly reduces the level of the H3K18la modification and H3 expression levels, suggesting that GCN5 acts as a lactylation “writer” [119]. Sun et al. reported that aminoacyl-tRNA synthetase (AARS)1/2 directly regulates methyltransferase-like protein 16 (METTL16) lactylation, indicating that AARS1/2 may be a lactyltransferase for METTL16 [120]. Mao et al. found that hypoxia induces AARS2 accumulation and that AARS2 lactylates PDHA1 at lysine 336, inactivating the pyruvate dehydrogenase complex and inhibiting OXPHOS [121]. Zhu et al. observed that AARS1 responds to intracellular lactate levels by translocating to the nucleus, interacting with the YAP–TEAD1 complex and lactylating both YAP and TEAD1 [122]. Zong et al. reported that AARS1 lactylates two residues, K120 and K139, in the p53 DBD to block p53 liquid‒liquid phase separation (LLPS) and DNA binding and induce the expression of its target genes, ultimately contributing to tumorigenesis [123]. In colorectal cancer, KAT8 acts as a pan-Kla writer, lactylating eukaryotic translation elongation factor 1 alpha 2 (eEF1A2), and the increased protein synthesis mediated by lactylation contributes to oncogenic adaptation [124]. In fibroblasts, KAT8 promotes the lactylation of latent transforming growth factor beta-binding protein 1 (LTBP1) at K752, increasing the protein levels of collagen I and collagen III in fibroblasts [125]. Lactic acid mediates the lactylation of Vps34 at K356/K781 via the acyltransferase KAT5/tat-interacting protein 60 (TIP60), promotes autophagic flux and endolysosomal transport, and leads to cancer progression [126]. The acetyltransferase GNAT13 functions as a lactyltransferase. It can catalyse Kla in vitro and lactylate the RNA polymerase subunit RpoA, thereby inhibiting the formation of oral biofilms [127]. HBO1 promotes H3K9la, and a subsequent pathway analysis revealed that pathways involved in carcinogenic mechanisms were enriched, which in turn promoted the malignant behaviours of cancer cells [128].

Similarly, delactylation requires “erasers”, enzymes that remove lactyl groups from substrates. Zhao et al. reported that histone deacetylases (HDACs) 1–3 are histone lysine delactylases [129]. Fan et al. reported that HDAC2 overexpression reduces H3K9 lactylation, inhibiting angiogenesis [130]. Studies have shown that under hypoxic conditions, SIRT3 reduces PDHA1 lactylation to promote OXPHOS [121]. Fan et al. found that SIRT3 is highly specific for the H4K16la site, in which the lactyl group is specifically removed from the H4K16 site [131]. Du et al. conducted a proteomic analysis and found that SIRT1 targets Kla proteins related to RNA metabolism, indicating a role for SIRT1 in controlling alternative splicing and RNA molecular stability; moreover, SIRT3 may counteract inflammatory responses and the harmful effects of alcohol by regulating the lactylation state of certain proteins [132]. In prostate cancer, SIRT1 mediates the delactylation of canopy FGF signalling regulator 3 (CNPY3), affecting its cellular localization and promoting lysosomal rupture to trigger pyroptosis [133]. In *Escherichia coli*, CobB can erase lactylation at PykF K382 (PykF K382la) to increase glycolysis and promote growth [134]. In colorectal cancer, HDAC1 inhibits H4K12la, promotes ferroptosis signalling, and reduces chemoresistance [135]. L-Lactic acid mainly promotes the lactylation of RBM15 at the K850 site, whereas HDAC3 is responsible for delactylation of this site, which disrupts the association between RBM15 and METTL3 and promotes the ubiquitin-mediated degradation of RBM15 [136]. Lactylation of METTL16 at the K229 site can be inhibited by SIRT2, thereby suppressing the ability of METTL16 to regulate cuproptosis and slowing the progression of copper-related cancer [120].

**Fig. 2:**
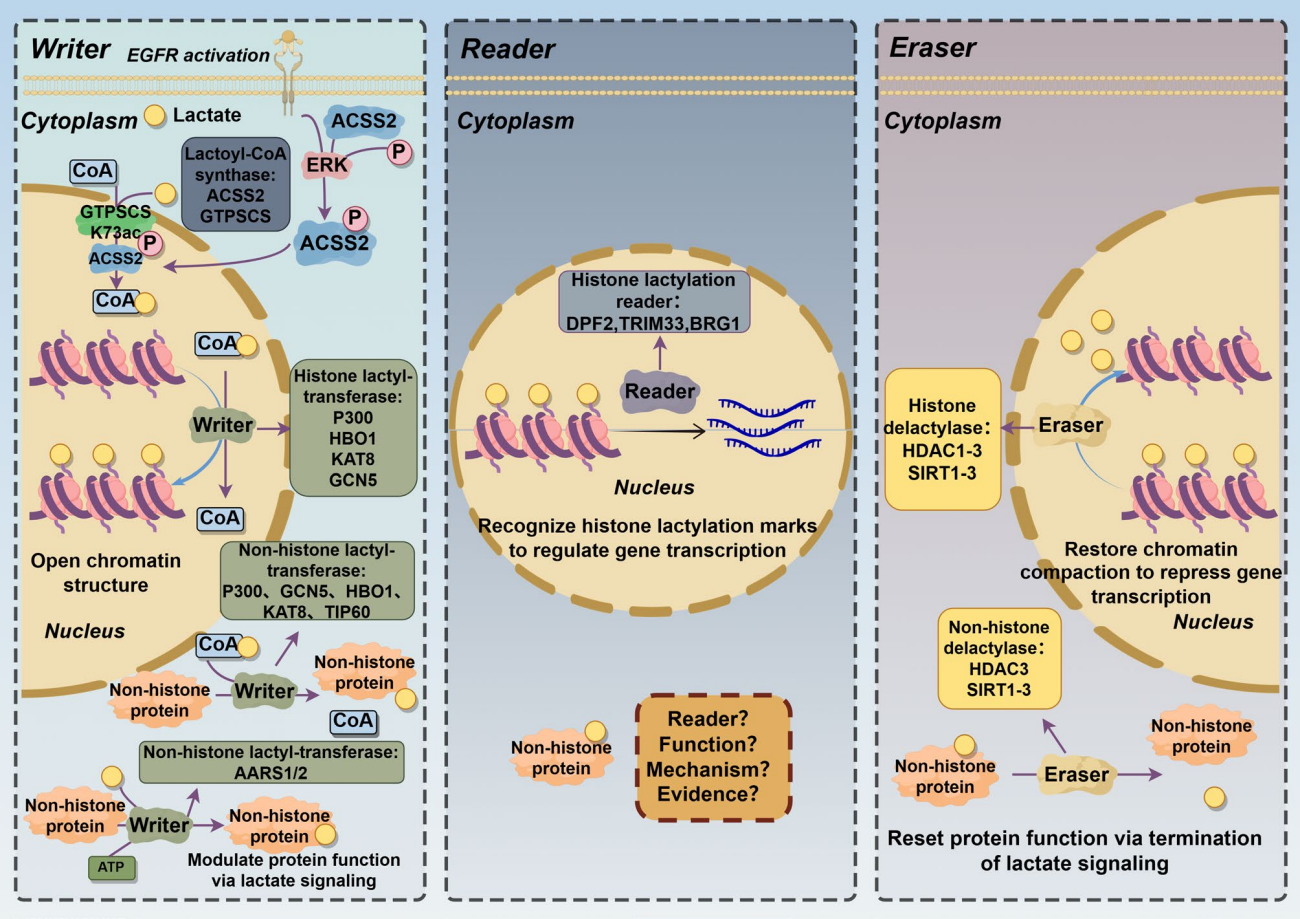
Molecular mechanisms of lactylation and associated enzymes

Lactylation functions through “readers”. In cervical cancer, Gui et al. observed that double PHD fingers 2 (DPF2) can bind to H3K14la on oncogene promoters, promoting cancer-related gene expression and cell survival [137]. The bromodomain TRIM33 binds to lactylated H3K18, promoting macrophage polarization to the M2 type and attenuating inflammatory responses [138]. The bromodomain-containing protein Brg1 interacts with H3K18la and facilitates its enrichment at mesenchymal–epithelial transition (MET)-related gene promoters, promoting induced pluripotent stem cell (iPSC) reprogramming [139, 140] (Fig. 2) (Table 2).

**Table 2:** Enzymes related to lactylation

Categories	Enzyme	Site	Functions	References
Lactyl donor synthetase	GTPSCS		synthesize lactyl-CoA	[114]
	ACSS2		synthesize lactyl-CoA	[115]
Writers	p300	H3K18, NCL K477	proliferation,invasion,drug resistance	[114, 116, 117]
	CBP	MRE11 K673	drug resistance	[118]
	GCN5	H3K18	promote repair	[119]
	AARS1	METTL16, YAP-TEAD1, p53 K120/K139	proliferation,tumorigenesis	[120, 122, 123]
	AARS2	METTL16, PDHA1 K336	proliferation	[120, 121]
	KAT8	eEF1A2, LTBP1 K752	tumorigenesis	[121, 125]
	KAT5/TIP60	Vps34 K356/K781	progression	[126]
	HBO1	H3K9la	tumorigenesis	[128]
Erasers	HDAC2	H3K9la	inhibit angiogenesis	[130]
	HDAC1	H4K12la	ferroptosis	[135]
	HDAC3	RBM15 K850	inhibit the proliferation and migration of tumor cells	[136]
	SIRT3	H4K16la	promote OXPHOS	[121, 131]
	SIRT2	METTL16 K229la	cuproptosis	[120]
	SIRT1	CNPY3 Kla	promote pyroptosis	[133]
Readers	DPF2	H3K14la	cell survival	[137]
	TRIM33	H3K18la	immunosuppression	[138]
	Brg1	H3K18la	progression, metastasis	[139]

A panoramic view of the enzymatic machinery that catalyzes the “writing,” “reading,” and “erasing” of lactyl groups on both histone and non-histone proteins. In the cytoplasm, L-lactate is first converted to lactyl-CoA by ACSS2 (and possibly GTPSCS). This high-energy donor is then imported into the nucleus, where two classes of lactyl-transferase “writers” operate:

- Histone lactyl-transferases—P300, GCN5, HBO1, KAT8, and TIP60—introduce lactylation marks such as H3K73la, generating open chromatin and activating transcription.
- Non-histone lactyl-transferases—P300/GCN5/HBO1/KAT8 as well as the newly identified AARS1/2—lactylate a broad spectrum of nuclear and cytoplasmic proteins in an ATP-dependent manner, modulating their function or stability.

Lactyl-lysine marks are specifically recognized by “reader” modules: DPF2, TRIM33, and BRG1 bind histone lactylation to recruit transcriptional complexes and propagate lactate-dependent signaling. Readers and downstream mechanisms for non-histone lactylation remain to be defined.

Removal of lactyl groups is executed by “eraser” delactylases. Histone delactylases, including HDAC1–3 and SIRT1–3, restore chromatin compaction and repress transcription, whereas non-histone delactylases (HDAC3 and SIRT1–3) reset protein function by terminating lactate signaling.

Thus, a dynamic lactylation cycle driven by the availability of lactyl-CoA integrates cellular metabolic state with epigenetic, transcriptional, and post-translational regulatory pathways.

### Lactylation in cancer

**Fig. 3:**
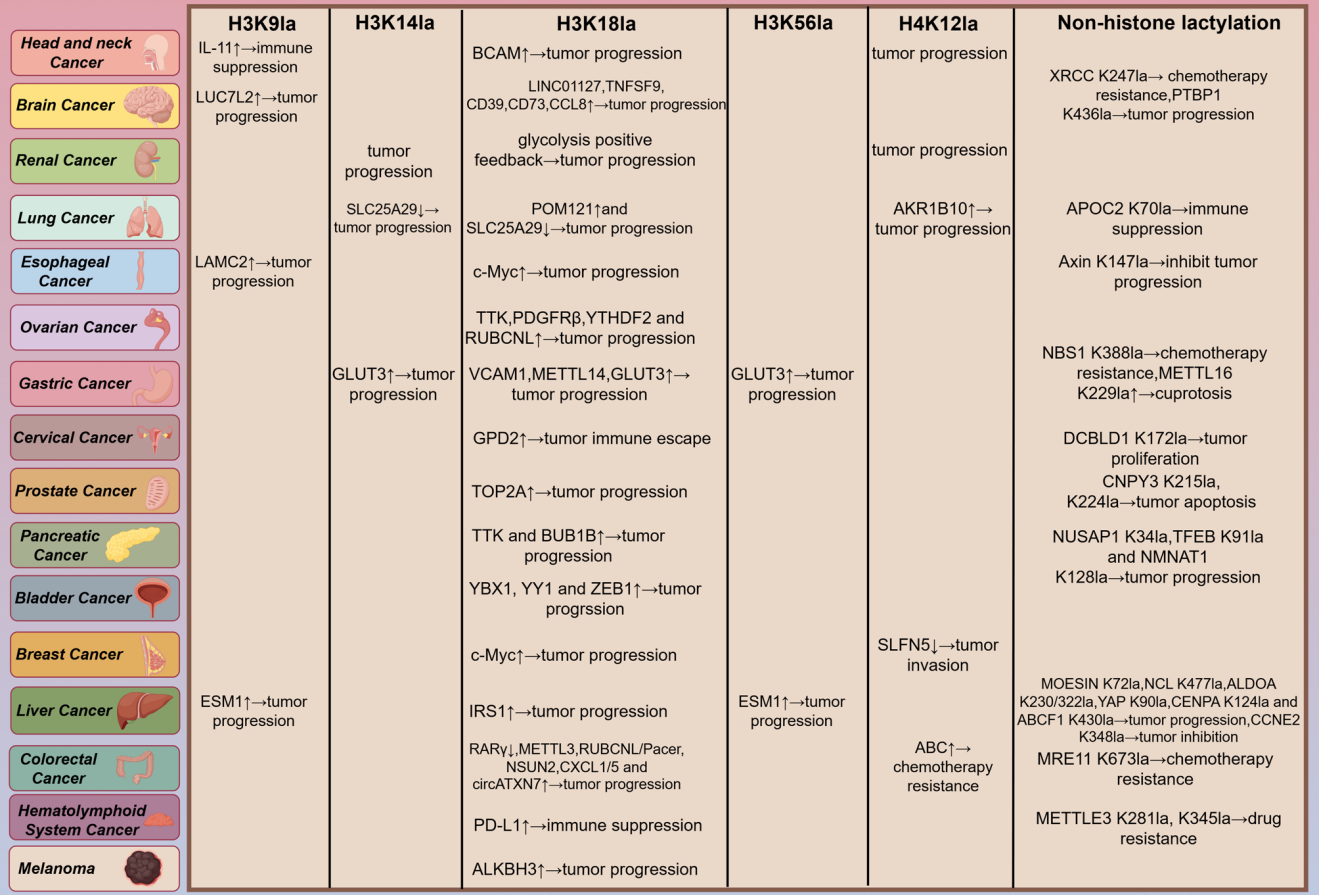
Lactylation in cancer

Emerging evidence links lactylation to multiple hallmarks of cancer, including immune evasion, altered metabolism and therapeutic resistance. Below, we have summarized the sites of action of lactylation in cancers of various organs throughout the body and its effects on cancer development (Fig. 3) (Table 3).

**Table 3:** Lactylation in tumors and its functions

Types of cancer	Site	Pathway	Effect	References
Gastric Cancer	H3K18la	VCAM1↑ → AKT-mTOR → CXCL1↑	recruitment of mesenchymal stem cells and M2 macrophages → tumor metastasis and high invasiveness	[91]
	H3K18la	LOX → TGF-β → IGF1↑ → lactate↑ → H3K18la → PD-L1↑	immune suppression	[141]
	H3K18la	METTL14↑ → ATF5↓ → inhibit WDR74/β-catenin	reduce the stemness of gastric cancer cells	[142]
	H3K9la, H3K18la, H3K56la	GLUT3↑ → LDHA↑ → H3K9la, H3K18la and H3K56la	tumor metastasis and high invasiveness	[143]
	NBS1 K388la	MRN complex↑	DNA repair → chemotherapy resistance	[144]
	METTL16 K229la	Cu → the binding of AARS1/2 protein to METTL16↑ → METTL16 K229la↑ → FDX1↑	DLAT lipoylation and copper—protein deposition → cuprotosis	[120]
Colorectal Cancer	H3K18la	METTL3↑ → METTL3la↑ → JAK/STAT3	continuous immunosuppressive activity of TIMs	[145]
	H3K18la	RUBCNL/Pacer → PI3K↑	proliferation and drug resistance	[146]
	H3K18la	RARγ↓ → TRAF6-IL-6-STAT3	chronic inflammation → tumor growth	[92]
	H3K18la	NSUN2↑ → NSUN2 K356la↑ → ENO1↑	promote glycolysis → promote tumorigenesis and drug resistance	[147]
	H3K18la	GPR37 → hippo signal pathway → LDHA↑ → H3K18la → CXCL1/5↑	promote tumor growth and angiogenesis	[148]
	H4K12la	SMC4↓ → lactate↑ → H4K12la → ABC↑	chemotherapy resistance	[149]
	H3K18la	circATXN7↑ → circATXN7 binds to the NF-κB p65 subunit	tumor—specific CTLs are sensitive to AICD; tumor drug resistance	[150]
	MRE11 K673la	unclear	promote DNA end resection and damage repair → Chemotherapy resistance	[118]
Pancreatic Cancer	H3K18la	TTK and BUB1B↑ → p300↑ → glycolysis positive feedback	drive the cell cycle and accelerate tumorigenesis	[122]
	NUSAP1 K34la	NUSAP1 K34la → NUSAP1↑ → combine with c-MYC and HIF-1α → LDHA↑ →	promote the growth and metastasis of tumors	[151]
	TFEB K91la	TFEB↑	maintain a high level of autophagy in cancer cells	[152]
	NMNAT1 K128la	NMNAT1 nuclear localization↑ → NAD↑ → Sirt1 activity↑	reduce cellular stress	[153]
Liver Cancer	H3K18la	SRSF10 → MYB↑ → GLUT1, HK1, LDHA↑ → H3K18la → M2 macrophage polarization	immune suppression and drug resistance	[94]
	H3K18la	PYCR1 → H3K18la → IRS1↑ → PI3K/AKT/mTOR, MAPK/ERK	proliferation and metastasis	[154]
	H3K9la, H3K56la	ESM1↑	proliferation and metastasis	[155]
	H3K56la	PDHX K488ac → interaction with E2 weakens → PDC↓ → glycolysis↑ → H3K56la	tumor progression	[156]
	MOESIN K72la	FGFR3,p53; TGF-β → SMAD pathway → Treg cells	immune suppression	[81]
	CCNE2 K348la	unclear	inhibit tumor growth and metastasis	[157]
	NCL K477la	MADD↑ → MEK/ERK pathway	tumorigenesis	[117]
	ALDOA K230/322la	interaction with DDX17 weakens → DDX17 activate	proliferation and metastasis	[158]
	YAP K90la	interaction with CRM1 weakens → YAP↑	antagonize sorafenib-induced ferroptosis	[159]
	CENPA K124la	CENPA↑ → CCND1, NRP2↑	proliferation and growth	[160]
	ABCF1 K430la	KDM 3A-H3K9me2-HIF 1α pathway	progression	[161]
Lung Cancer	H3K18la	POM121 → nuclear import of MYC → PD-L1↑	immune suppression	[162]
	H3K14la	Mir100hg → ALDOA↑ → H3K14la → expression of metastasis-related genes	metastasis	[163]
	H4K12la	AKR1B10↑ → LDHA↑ → H4K12la → CCNB1↑	tumor metastasis and drug resistance	[164]
	H3K14la, H3K18la	SLC25A29↓	increased proliferation and migration and decreased apoptosis of endothelial cells	[165]
	APOC2 K70la	FFA↑ → Treg cells↑	immunotherapy resistance and tumor metastasis	[97]
Brain Cancer	H3K9la	LUC7L2↑ → MLH1↓	inhibit MMR and tumor resist to TMZ	[166]
	H3K18la	CD39,CD73,CCL8↑	immune escape	[167]
	H3K18la	LINC01127↑ → MAP4K4↑ → JNK pathway	tumor growth	[168]
	H3K18la	TNFSF9↑	macrophage M2 polarization and immune suppression	[95]
	XRCC K247la	ALDH1A3↑ → lactate↑ → XRCC K247la → the nuclear import of XRCC↑	DNA damage repair and tumor chemoresistance	[169]
	PTBP1 K436la	the interaction between PTBP1 and TRIM21↓ → PTBP1↑ → PFKFB4↑ → glycolysis	tumor development	[170]
Melanoma	H3K18la	ALKBH3↑ → SP100A↓ → PML↓	tumor growth and metastasis	[171]
Esophageal Cancer	H3K9la	LAMC2↑ → PI3K/AKT pathway → VEGFA↑	tumor angiogenesis → tumor growth and metastasis	[172]
	H3K18la	AP001885.4 → lactylation↑ → c-MYC↑	tumor proliferation	[173]
	Axin K147la	Axin↓	inhibit tumor growth	[174]
Breast Cancer	H3K18la	c-MYC↑ → SRSF10↑ → Alternative splicing of MDM4 and Bcl-x	tumorigenesis and tumor development	[175]
	H4K12la	SLFN5↓ → ZEB1↓	tumor invasion	[176]
	H3K18la	KCNK1↑ → LDHA1 → H3K18la↑	tumor development	[177]
Cervical Cancer	H3K18la	GPD2↑ → M2 macrophage polarization	tumor immune escape	[96]
	DCBLD1 K172la	HIF-1α → DCBLD1↑ → G6PD↑	tumor proliferation	[178]
Renal Cancer	H3K18la	Inactivated VHL → HIF → lactate → H3K18la → glycolysis positive feedback	tumor angiogenesis	[179]
	H3K14la,H3K18la, H3K56la	FKBP10 → LDHA-Y10p → glycolysis → H3K14la,H3K18la, H3K56la	proliferation and migration	[180]
Prostate Cancer	H3K18la	DNA topoisomerase II alpha (TOP2A)↑ → LDHA↑ → lactate → lactylation	tumor progression	[181]
	CNPY3 K215la, K224la	GBA → SIRT1↑ → Delactylation of CNPY3	apoptosis	[133]
Bladder Cancer	H3K18la	circXRN2 → LATS1↑ → Hippo pathway → LCN2, H3K18la↓	suppress tumor progression	[182]
	H3K18la	ZEB1↑	tumor migration	[183]
	H3K18la	YBX1, YY1↑	drug resistance	[184]
Ovarian Cancer	H3K18la	M2 macrophage polarization → CCL-18↑	growth and migration	[185]
	H3K18la	TTK, PDGFRβ, YTHDF2 and RUBCNL↑	tumor growth	[186]
Head and Neck Cancer	H3K9la	IL-11↑ → JAK2/STAT3 pathway → Dysfunction of CD8^+^ T cells	immune suppression	[187]
	H4K12la	BRAFV600E → H4K12la → The expression of proliferation-related genes	progression	[188]
	H3K18la, H3K27la	BCAM↑	invasion, angiogenesis, and chemoresistance	[189]
Hematolymphoid System Cancer	H3K18la,METTLE3K281la, K345la	lactate → H3K18la, METTLE3K281,345la → METTLE3↑	drug resistance	[190]
	H3K18la	STAT5↑ → H3K18la → PD-L1↑	immune suppression	[191]

#### Lactylation in gastric cancer

Due to the Warburg effect, lactate accumulates in large amounts, promoting the TIP60-mediated lactylation of NBS1 at K388, which in turn promotes the formation of the MRN complex to aid in DNA repair and induce tumor chemoresistance [144]. In gastric cancer, copper levels are significantly increased, and copper ions may bind to the metallothionein cluster-binding region of AARS1/2 proteins, causing conformational changes in AARS1/2 and increasing their binding to METTL16, which promotes the lactylation of METTL16 at K229 [192]. This lactylation upregulates the m6A modification of the ferredoxin 1 (FDX1) mRNA, promoting FDX1 production and inducing DLAT lipidation and copper protein deposition to ultimately lead to a unique type of cell death known as cuproptosis [120]. Excess lactate promotes H3K18la, upregulating vascular cell adhesion molecule 1 (VCAM1), which increases AKT–mTOR-mediated chemokine (C-X-C motif) ligand 1 (CXCL1) expression and leads to the recruitment of mesenchymal stem cells and M2-type macrophages, ultimately resulting in immunosuppression in the TME [91]. Glucose transporter 3 (GLUT3) expression is significantly increased in tumor tissues and promotes lactate production and lactylation at H3K9, H3K18, and H3K56 by regulating LDHA, which in turn promotes the EMT, leading to tumor metastasis and increased invasion [143]. Cancer-associated fibroblasts (CAFs) secrete lysyl oxidase (LOX) to activate the TGF-β signalling pathway, leading to increased expression of insulin-like growth factor 1 (IGF1) and promoting gastric cancer cell migration, EMT, and glycolysis. Glycolysis produces lactate, promoting lactylation. H3K18la enrichment at the PD-L1 promoter region promotes PD-L1 transcription, ultimately leading to immunosuppression in tumor tissues [141]. Histone H3K18la upregulates METTL14 expression, promoting the m6A modification of the ATF5 mRNA and leading to ATF5 downregulation, thereby inhibiting the WD repeat domain 74 (WDR74)/β-catenin axis and reducing gastric cancer cell stemness [142]. Lactylation of NOP2/Sun RNA methyltransferase 2 (NSUN2) at the K508 site increases its activity, stabilizes the glutamate-cysteine ligase catalytic subunit (GCLC) m5C mRNA, upregulates GCLC expression, increases the intracellular GSH level, and leads to the resistance of cancer cells to ferroptosis [193].

Lactate plays important roles in metabolic regulation, gene expression, the tumor microenvironment, and drug resistance in gastric cancer (GC) [194, 195]. In-depth research into the mechanism of lactate activity and the development of lactate-targeted therapeutic strategies are expected to provide new insights for the early diagnosis, treatment, and prognostic evaluation of gastric cancer (GC). However, the regulatory mechanism of histone lactylation in GC remains unclear [91]. How AARS1/2 functions as a lactate transferase and whether other factors are involved in supporting or regulating the enzymatic activity of AARS1/2 remain to be further explored [120]. Further research is needed to elucidate the pathways through which lactylation-related genes influence immune cell infiltration and genomic instability in gastric cancer (GC) [196]. The clinical relevance of AARS1 in various gastric cancer (GC) subtypes and other types of malignancies remains to be elucidated. Additionally, given the critical role of AARS1 in tRNA aminoacylation and protein synthesis, further research is necessary to explore the potential of targeting the lactyltransferase activity of AARS1 for the treatment of GC and other human malignancies [122].

#### Lactylation in colorectal cancer

Lactate accumulation in the tumor microenvironment promotes H3K18 lactylation, which in turn increases the expression of METTL3 in tumor-infiltrating myeloid cells (TIMs). Lactylation sites are also detected in METTL3, helping it capture m6A-modified target RNAs, activating the downstream JAK/STAT3 regulatory axis and maintaining the continuous immunosuppressive activity of TIMs [145]. H3K18 lactylation increases Rubicon-like autophagy enhancer (RUBCNL)/Pacer transcription, which then interacts with beclin 1 (BECN1) to promote autophagosome maturation and mediate the recruitment and function of class III PI3K, aiding in cancer cell proliferation and survival in hypoxic environments and weakening the efficacy of bevacizumab treatment [146]. MRE11 is an important homologous recombination (HR) protein. After DNA damage occurs, CBP acetyltransferase interacts with MRE11, lactylating MRE11 at K673, promoting its binding to DNA, and promoting DNA end resection and damage repair, and the over-enhanced HR leads to tumor chemoresistance [118]. G-protein-coupled receptor 37 (GPR37) activates the Hippo pathway, promoting LDHA expression and glycolysis, leading to increased H3K18la expression, the upregulation of CXCL1 and CXCL5 expression, and ultimately tumor growth and angiogenesis [148]. Lipopolysaccharide (LPS) produced by gut bacteria induces lactylation of the LINC00152 promoter, increasing its expression and reducing its binding to the negative regulator yin yang 1 (YY1). LINC00152 upregulation reduces IL-8 and TNF-α expression, inhibiting the inflammatory response through the NF-κB pathway and promoting tumor growth and migration [194]. Structural maintenance of chromosome 4 (SMC4) attenuation promotes glycolysis, increasing lactate production, leading to lactylation of H4K12 at the ABC transporter gene promoter, increasing ABC protein expression, and ultimately leading to chemoresistance [149]. Tumor-derived lactate promotes H3K18 lactylation, inhibits retinoic acid receptor gamma (RARγ) gene transcription in macrophages, and activates tumor necrosis factor receptor-associated factor 6 (TRAF6)-IL-6-STAT3 signalling, and sustained chronic inflammation promotes tumor growth [92]. Intratumoral bacteria such as *E. coli* promote lactate production and retinoic acid-inducible gene 1 (RIG-I) lactylation, which inhibits NF-κB recruitment to the Nlrp3 promoter, thus mediating M2 macrophage polarization. This process promotes the immunosuppressive activity of Treg cells and inhibits the cytotoxic activity of CD8^+^ T cells [93]. In tumor-specific CTLs, circATXN7 transcription is activated by H3K18 lactylation, and circATXN7 binds to the NF-κB p65 subunit, masking the p65 nuclear localization signal motif and sequestering it in the cytoplasm, which leads to tumor-specific CTL sensitivity to activation-induced cell death (AICD) and tumor resistance [150]. Aldolase B (ALDOB) activates pyruvate dehydrogenase kinase 1 (PDK1), inducing lactate production and secretion, promoting CEA cell adhesion molecule 6 (CEACAM6) expression, and promoting colorectal cancer (CRC) cell proliferation and chemoresistance [197]. Lactate accumulation in CRC cells activates NSUN2 transcription through histone H3K18 lactylation and induces NSUN2 lactylation at Lys356 (K356), facilitating the capture of target RNAs. NSUN2 and YBX1 together promote the expression of the core glycolytic enzyme enolase 1 (ENO1), forming a positive feedback loop and ultimately promoting tumorigenesis and drug resistance [147]. The K412la modification of HDAC1 in the colorectal region renders cancer cells insensitive to ferroptosis [198].

Previous studies have shown that lactylation plays crucial roles in multiple processes of colorectal cancer, including its occurrence, development, metastasis, immune evasion, and response to treatment [118, 145, 150]. However, whether p300 and HDAC1 are involved in other histone lactylation modifications is unclear. Future research may provide a theoretical basis for the development of histone lactylation inhibitors [135]. Further research is also needed to explore the role of epigenetics in ferroptosis and to investigate whether other histone lactylation modifications affect the expression of ferroptosis-related genes [135]. An exploration of the significance of nonlysine lactylation sites in regulating the function of myeloid cells in the TME is also possible [145]. Further research is needed in the future to fully understand the cooperation between lactylation and acetylation in the epigenetic regulation of stemness-related genes, which will provide comprehensive insights into the relationships among metabolism, epigenetics, and cell fate determination [199].

#### Lactylation in pancreatic cancer

H3K18la is enriched at the promoters of the mitotic checkpoint regulators TTK and BUB1B, activating their transcription. TTK and BUB1B increase p300 expression, promoting glycolysis to form a positive feedback loop that drives the cell cycle and accelerates tumorigenesis [122]. Lactylation of the transcription factor transcription factor EB (TFEB) at K91 prevents TFEB from interacting with the E3 ubiquitin ligase WW domain-containing protein 2 (WWP2), inhibiting TFEB ubiquitination and proteasomal degradation, maintaining high levels of autophagy in cancer cells, and promoting cancer cell survival, growth and proliferation [152]. Lactate inhibits the degradation of the nucleolar and spindle-associated protein 1 (NUSAP1) protein through NUSAP1 K34la, leading to its upregulation. NUSAP1 can bind to c-MYC and hypoxia-inducible factor 1α (HIF-1α) to form a transcriptional regulatory complex localized at the promoter region of LDHA, which promotes the expression of LDHA. LDHA further promotes glycolysis, forming a positive feedback loop that facilitates tumor growth and metastasis [151]. Lactate lactylates nicotinamide mononucleotide adenylyltransferase 1 (NMNAT1) at K128, possibly increasing its nuclear localization and maintaining NMNAT1 activity to prevent NAD depletion, activate Sirt1, reduce sustained cellular stress, and promote cell survival [153].

In pancreatic cancer, lactylation regulates key molecules and pathways at multiple levels and significantly affects processes such as tumor cell proliferation, metabolism, autophagy, and survival [116, 122, 152]. Further research is still needed to determine whether other acylation events mediated by p300 are involved in malignant phenotypes [116]. Studies are needed to clarify the precise effects of histone lactylation at specific sites, such as H3K14 and H3K18, on chromatin structure, as well as the subsequent regulation of gene transcription mechanisms [115]. The involvement of multiple writers and erasers in both lactylation and acetylation leads to complications, which poses challenges for the development of HAT and HDAC inhibitors for Kla-targeted therapy [200]. Researchers should focus on the isolation and detailed characterization of different cell subpopulations in the tumor microenvironment to explore potential therapeutic targets [201].

#### Lactylation in liver cancer

Lactate induces the EMT through the lactylation of MOESIN at K72, promoting FGFR3 signalling and p53 signalling, and regulating the cell cycle. MOESIN lactylation at K72 promotes its interaction with the TGF-β receptor, regulating the SMAD signalling pathway, inducing Treg generation, and leading to immunosuppression [81]. SIRT3 delactylates cyclin CCNE2 at K348, inhibiting tumor growth and metastasis [157]. In intrahepatic cholangiocarcinoma (iCCA), nucleolin NCL lactylation at K477 upregulates MAP kinase-activating death domain-containing protein (MADD) through an RNA splicing-dependent mechanism, activating the classical MEK/ERK pathway and promoting tumorigenesis [117]. Centromere protein centromere protein A (CENPA) lactylation at K124 promotes CENPA transcriptional activation, upregulating its expression. CENPA then cooperates with YY1, driving the expression of cyclin D1 (CCND1) and neuropilin 2 (NRP2) and promoting hepatocellular carcinoma proliferation and growth [160]. Serine- and arginine-rich splicing factor 10 (SRSF10) interacts with the 3'-untranslated region of MYB, increasing MYB RNA stability. MYB upregulates key glycolytic enzymes, including glucose transporter 1 (GLUT1), hexokinase 1 (HK1), and LDHA, leading to increased lactate levels and further promoting MYB generation. Lactate induces histone H3K18 lactylation, promoting M2 macrophage polarization and subsequent immunosuppression and drug resistance [94]. Hepatocellular carcinoma aldolase A (ALDOA) lactylation at K230/322 weakens the tight binding between ALDOA and DEAD-box helicase 17 (DDX17), increasing the ability of DDX17 to maintain liver cancer stem cell stemness and leading to tumor proliferation and metastasis [158]. Hepatocellular carcinoma pyrroline-5-carboxylate reductase 1 (PYCR1) upregulation promotes glycolysis and increases insulin receptor substrate 1 (IRS1) histone H3K18 lactylation, increasing IRS1 expression, activating the PI3K/AKT/mTOR and MAPK/ERK pathways, and promoting tumor proliferation and metastasis [154]. H3K9 lactylation and H3K56 lactylation activate endothelial cell-specific molecule 1 (ESM1) transcription in HCC cells, promoting cancer cell proliferation and metastasis [155]. Pyruvate dehydrogenase complex component X (PDHX) is acetylated by p300 at K488, weakening its interaction with dihydrolipoamide acetyltransferase (E2), disrupting mitochondrial pyruvate dehydrogenase complex (PDC) assembly and inhibiting its activation, which lead to increased glycolysis, H3K56 lactylation-mediated gene expression, and tumor progression [156]. YAP lactylation at K90 weakens its binding affinity with chromosomal maintenance 1 (CRM1), promoting YAP nuclear accumulation and overactivation in HCC cells and leading to cancer cell resistance to sorafenib-induced ferroptosis [159]. In hepatocellular carcinoma (HCC), the level of ABCF1 lactylated at K430 (ABCF1 K430la) increases, its degradation decreases, and it upregulates lysine demethylase 3 A (KDM3A), activating the KDM3A–H3K9me2–HIF1α axis, which promotes the malignant progression of HCC [161]. In hepatocellular carcinoma, c-MYC activates STAT3 through direct binding and simultaneously increases the phosphorylation of STAT3 downstream of endoplasmic reticulum stress (ERS) induced by glucose-regulated protein 78 (GRP78). After lactylation at the H3K18 site of GP73, its expression is upregulated. STAT3 can enhance the proangiogenic function of GP73, leading to tumor progression [202].

Overall, lactylation plays important roles in multiple key processes in tumor cells, including metabolic reprogramming, cell cycle regulation, stemness maintenance, invasion, metastasis, and immune evasion [81, 157, 158]. The impact of lactylation on immune cells remains to be further discussed [81]. Moreover, targeted therapies using FGFR, IDH, and ErbB2 inhibitors have shown limited benefits in clinical practice, highlighting the necessity of an in-depth multiomics exploration into the prospects of treating intrahepatic cholangiocarcinoma (iCCA) [117]. In terms of lactate-regulating enzymes, LDHB is distributed mainly in the brain and heart, where it catalyses the conversion of lactate to pyruvate, and its expression is downregulated during liver injury. However, current research has focused on LDHA; thus, the mechanism by which LDHB regulates lactate in hepatocytes remains to be explored [203]. Additionally, through NRF2 activation, lactylation can promote the survival of cancer cells under oxidative stress conditions, but whether similar lactylation-driven processes exist in other types of cancer remains to be studied [204].

#### Lactylation in lung cancer

In non-small cell lung cancer (NSCLC), increased H3K18 lactylation activates the transcription of pore membrane protein 121 (POM121), enhancing MYC nuclear transport. MYC binds to the CD274 promoter, promoting PD-L1 expression and leading to tumor immunosuppression [162]. Hypoxia induces SRY-box transcription factor 9 (SOX9) lactylation, increases glycolysis, and promotes cell stemness, migration, and NSCLC invasion [205]. In lung adenocarcinoma (LUAD), elevated basic leucine zipper and W2 domain 2 (BZW2) levels promote glycolysis, increased lactate production, and increased isocitrate dehydrogenase 3 gamma (IDH3G) lactylation, leading to tumor cell proliferation and migration and the inhibition of apoptosis [206]. Lactate lactylates apolipoprotein C2 (APOC2) at K70, stabilizing it and causing free fatty acid (FFA) release, Treg cell accumulation, immunotherapy resistance, and tumor metastasis [97]. AKR1B10 upregulation increases LDHA expression, promotes glycolysis, increases H4K12 lactylation levels, promotes cyclin B1 (CCNB1) transcription, accelerates DNA replication, promotes the cell cycle, and leads to tumor metastasis and drug resistance [164]. Fargesin (FGS), a drug targeting pyruvate kinase M2 (PKM2), regulates aerobic glycolysis, inhibits H3la, and thereby inhibits tumor proliferation and metastasis [207]. In lung adenocarcinoma (LUAD), H3K14la and H3K18la accumulate at the SLC25A29 promoter region, downregulating SLC25A29 expression, increasing endothelial cell proliferation and migration, and reducing apoptosis [165]. Exosomal Mir100hg derived from cancer stem cells (CSCs) binds to HNRNPF and HNRNPA2B1, facilitating its transportation to non-CSCs. It then upregulates the expression of ALDOA, resulting in the lactylation of H3K14 and promoting the transcription of 169 metastasis-related genes. Eventually, these changes increase metastatic potential of non-CSC lung cancer cells [163]. Lactic acid directly activates the transcription of Cthrc1 in CAFs through H3K18la, thus forming a positive CTHRC1/glycolysis/H3K18la feedback loop that drives resistance to EGFR-tyrosine kinase inhibitors (EGFR-TKIs) [208].

In summary, lactylation participates in the proliferation, metastasis, metabolic reprogramming, immune escape, and therapeutic resistance of non-small cell lung cancer (NSCLC) through multiple mechanisms, such as epigenetic regulation, modifications of protein function, and intercellular signal transmission [97, 162–164]. Hypoxia promotes the lactylation of SOX9, which increases glycolysis to facilitate the generation, migration, and invasion of non-small cell lung cancer (NSCLC) stem cells. Inhibiting hypoxia thus emerges as a novel therapeutic approach for NSCLC, but extensive research is still needed to translate this conclusion into clinical applications [205]. LKB1 promotes cellular senescence and regulates telomerase through histone lactylation. However, more studies are needed to clarify the impact of histone lactylation on the transcription of other genes, as well as the crosstalk between cellular senescence and other biological cancer processes [209]. While the current understanding of the role of lactate in the tumor microenvironment has advanced to some extent, more comprehensive research is needed in the future. Specifically, the effect of nonhistone lactylation on gene transcription in Treg cells remains unknown [210].

#### Lactylation in brain cancer

In glioblastoma (GBM), tumor-derived factors activate PERK–ATF4 signalling to induce GLUT1 expression in monocyte-derived macrophages (MDMs), promote lactate production, increase lactylation levels, regulate IL-10 expression, and induce tumor immunosuppression [211]. In glioblastoma, ALDH1A3 expression is increased, and ALDH1A3 interacts with PKM2 to promote lactate accumulation and increase XRCC lactylation at K247, which changes it from negatively charged to neutral, increasing its affinity for nuclear transport proteins, promoting the nuclear import of XRCC, inducing DNA damage repair, and leading to tumor chemoresistance [169]. H3K9 lactylation occurs at the LUC7-like protein 2 (LUC7L2) promoter to promote its transcription and mediate MLH1 intron 7 retention; MLH1 is subsequently downregulated, and NMD recognizes intron 7 of PTC, leading to MLH1 mRNA degradation, the inhibition of mismatch repair (MMR), and the induction of temozolomide (TMZ) resistance in tumors [166]. In glioblastoma (GBM), mitogen-activated protein kinase 6 pseudogene 4 (MAPK6P4) encodes P4-135aa, which phosphorylates Kruppel-like factor 15 (KLF15) at S238 to stabilize it and promote its entry into the nucleus, promoting LDHA transcription, increasing VEGFR2 and VE-cadherin lactylation, upregulating their expression, and promoting tumor angiogenesis [212]. Increased H3K18 lactylation increases CD39, CD73, and CCL8 promoter activity, increasing their generation and leading to tumor immune escape [167]. H3K18 lactylation upregulates LINC01127 expression, promotes MAP4K4 expression, activates the JNK pathway, and ultimately promotes tumor growth [168]. Lactylated histones interact with CBX3, promoting CBX3 binding to the histone acetyltransferase EP300, with EP300 substrate specificity favouring lactyl-CoA and promoting lactylation [213]. Polypyrimidine tract-binding protein 1 (PTBP1) is a central regulator of RNA processing. In glioma stem cells (GSCs), PTBP1 lactylation at K436 weakens its interaction with TRIM21, inhibiting PTBP1 degradation and enhancing its RNA-binding ability to stabilize the 6-phosphofructo-2-kinase (PFKFB4) mRNA, further increase glycolysis, and promote tumor development [170]. Lactate absorbed by macrophages activates the MCT1/H3K18la signalling pathway, increasing TNFSF9 expression and leading to macrophage M2 polarization and tumor immunosuppression [95]. Hypoxia induces the upregulation of HIF-1α, which promotes glycolysis. Through the modification of H3K18la, it upregulates YTH domain-containing family protein 2 (YTHDF2), increases the interaction between YTHDF2 and BNIP3, and promotes BNIP3-dependent mitophagy-mediated metabolic reprogramming, leading to the proliferation and invasion of glioma cells [214].

Recent studies have shown that lactylation is involved in tumor immune escape and microenvironment remodelling. It can induce the metabolic reprogramming of macrophages and activate immune checkpoint molecules and can also promote angiogenesis and tumor proliferation through signalling pathways. The level of lactylation in the brain is believed to regulate neuronal excitability, inflammation, and development, among other processes. By regulating metabolism, epigenetics, and the immune microenvironment, lactylation results in the formation of a multilevel network for the maintenance of malignant phenotypes and therapeutic resistance in brain cancer [167, 211–213, 215]. In-depth research on the mechanism of action of lactate and lactylation of related proteins in the brain must be conducted, especially in the context of stroke and cognitive impairment, to provide new insights and ideas for exploring the connection between protein lactylation and brain neural function [215]. A more comprehensive analysis of the effects of lactylation on different subtypes of cells is needed; for example, since microglia exhibit functional heterogeneity, studies can be performed to investigate the role of macrophages derived from microglia with different functions in tumors after being affected by lactate [211]. Current research focuses on the interaction between tumor cells and microglia/macrophages, but lactate is also produced by other cell types in the brain, such as astrocytes, which play a key role in brain tumor biology and thus deserve further study [213].

#### Lactylation in melanoma

EP300 promotes YTHDF2 promoter lactylation, promoting YTHDF2 expression. YTHDF2 recognizes the m6A-modified PER1 and TP53 mRNAs, promoting their degradation and leading to tumorigenesis [216]. H3K18 lactylation increases alpha-ketoglutarate-dependent dioxygenase homologue 3 (ALKBH3) expression, leading to SP100 nuclear antigen subtype A (SP100A) m1A demethylation, the downregulation of SP100A expression, and decreased formation of tumor-suppressive promyelocytic leukaemia protein (PML) condensates that induce the loss of tumor suppression and tumor growth and metastasis [171].

Studies have shown that lactylation modifications target key transcriptional regulatory factors (such as EP300) and histone loci (such as H3K18) and can interfere with the function of tumor suppressor genes through the activation of RNA-binding proteins (YTHDF2) or RNA demethylases (ALKBH3) via the m⁶A and m^1^A RNA modification pathways, respectively, thereby promoting tumorigenesis and development [171, 216]. These findings not only expand the functional dimensions of lactylation but also reveal the core role of crosstalk between epigenetic modifications and RNA modifications in tumors, providing an important theoretical basis for the development of combined therapeutic strategies targeting lactylation and related RNA modifications. EP300-mediated lactylation plays a role in the transcriptional regulation of YTHDF2 in ocular melanoma, and further exploration can be conducted to determine whether other modifications written by EP300 also affect this process [216]. Recent studies have shown that lactylation is associated with the occurrence and development of melanoma, but studies with more samples are needed to confirm its clinical relevance [217]. Crizotinib can act as a CD147–MCT1 inhibitor to regulate lactate transport in cancer cells and macrophages. In the future, a more comprehensive characterization of other immune components, such as dendritic cells, regulatory T cells, and MDSCs, will help clarify the broader immunological impacts of crizotinib [218].

#### Lactylation in oesophageal cancer

Serine hydroxymethyltransferase 2 (SHMT2) lactylation increases its stability and levels, increasing MTHFD1L expression. MTHFD1L regulates ERK5 signalling, promoting tumor proliferation, drug resistance, and migration [219]. Hypoxia-induced H3K9la is enriched at the LAMC2 promoter, increasing its expression, activating the PI3K/AKT pathway, upregulating VEGFA expression, promoting angiogenesis, and promoting tumor proliferation and metastasis [172]. Hypoxia induces Axin protein lactylation at K147 to promote Axin ubiquitination and subsequent degradation, suppressing the role of Axin1 in promoting glycolysis and cell stemness, and inhibiting tumor growth [174]. AP001885.4 promotes H3K18la and protein panlactylation, upregulating c-MYC and leading to tumor proliferation [173].

In oesophageal cancer, lactylation regulates the degradation of metabolic enzymes to drive malignant tumor phenotypes [219]; in addition, histone lactylation regulates tumor angiogenesis and tumor cell proliferation [172, 173], and nonhistone lactylation has a bidirectional regulatory effect, as, in some cases, lactylation can have a tumor-suppressive effect [174]. Lactylation has multiple regulatory effects on tumor metabolic reprogramming, angiogenesis, proliferation, and stemness maintenance by targeting metabolic enzymes (such as SHMT2), histone loci (such as H3K9 and H3K18), and signalling molecules (such as Axin) and through the bidirectional effects of “promoting cancer” and “suppressing cancer” [172]. In current research, attention is often focused only on glucose uptake and lactate production in hypoxia-induced metabolic reprogramming. However, cells can utilize lipids and amino acids to provide energy, and these metabolites often also affect histone modifications, such as histone acetylation, succinylation, and methylation, which are equally worthy of research. Similarly, in the future, more histone lactylation sites need to be studied under hypoxic conditions to ensure the comprehensiveness of the research[172].

#### Lactylation in breast cancer

H3K18la is enriched at the c-MYC promoter, promoting its expression. c-MYC upregulates serine/arginine splicing factor 10, leading to MDM4 and B-cell lymphoma-extra large (Bcl-x) alternative splicing and subsequent tumorigenesis and tumor development [175]. Potassium two-pore domain channel subfamily K member 1 (KCNK1) is upregulated in breast cancer, where it binds and activates LDHA1 to increase glycolysis and lactate accumulation, and promote H3K18 lactylation and downstream signalling pathways, leading to tumor development [177]. H4K12la binds to the schlafen family member 5 (SLFN5 promoter) and inhibits SLFN5 expression, reducing its binding to the zinc-finger E-box-binding homeobox 1 (ZEB1) promoter, inhibiting ZEB1 transcription, and weakening its inhibition of tumor invasion [176]. HDAC2 promotes the delactylation of METTL3 at the K27 site and facilitates the interaction between METTL3 and Wilms' tumor-1 associated protein, leading to the upregulation of the N6-methyladenosine (m^6^A) level in DNA damage repair [220].

Lactylation is intimately involved in tumor progression through multiple mechanisms: it regulates the expression of oncogenes and tumor suppressor genes by targeting histones (H3K18 and H4K12) [175, 176], amplifies malignant phenotypes through a metabolism-modifying positive feedback loop [177], and participates in repairing DNA damage by regulating RNA methylation via delactylation [221]. The interaction between KCNK1 and LDHA upregulates lactylation at the H3K18 site, but how their interaction mode at the 3D level affects LDHA remains to be further explored [177]. Although lactate is generally regarded as a tumor-promoting metabolite in the TME, it may also exert tumor-suppressive effects under different environmental conditions. Elucidating its precise role in different stages and subtypes of breast cancer remains a key research area. Similarly, although lactylation affects protein function and stability, the identification of specific protein targets and the mechanisms by which lactylation contributes to the pathogenesis of breast cancer still need to be fully elucidated [222]. In terms of treatment, the metabolic heterogeneity among breast cancer subtypes requires a nuanced understanding of lactate and lactylation dynamics. Therefore, determining whether their roles differ across major subtypes is a key research direction for the future [222].

#### Lactylation in cervical cancer

HIF-1α enrichment at the discoidin, CUB and LCCL domain-containing protein 1 (DCBLD1) promoter region increases DCBLD1 mRNA expression, and DCBLD1 lactylation at K172 stabilizes DCBLD1 expression. DCBLD1 overexpression inhibits G6PD autophagic degradation, activating the pentose phosphate pathway and promoting tumor proliferation [178]. HPV16 E6 inhibits G6PDla, promoting G6PD dimer formation, increasing its activity, activating the pentose phosphate pathway, and leading to tumor cell proliferation [223]. Lactate upregulates H3K18la expression, increases GPD2 expression, promotes macrophage M2 polarization, and leads to tumor immune escape [96].

Studies have shown that lactylation is intimately involved in tumor metabolic reprogramming and immune evasion through multiple mechanisms: stabilizing metabolic regulatory proteins (such as DCBLD1) to activate the pentose phosphate pathway, increasing metabolic enzyme activity by targeting viral proteins (HPV16 E6), and driving the M2 polarization of macrophages through histone modifications. These findings not only expand the functional dimensions of lactylation in tumor metabolism and immune regulation but also reveal its cross-links with viral carcinogenesis and hypoxic adaptation, providing an important theoretical basis for the development of tumor therapeutic strategies targeting lactylation and related metabolic–immune pathways [96, 178, 223]. DPF2 recognizes lactylated histones in cervical cancer, thereby mediating a putative mechanism of oncogene transcription in cancer cells, and further research can be conducted to explore whether this mechanism functions in other models, including cancer metastasis, chemoresistance and DNA damage repair [137].

#### Lactylation in renal cancer

In clear cell renal cell carcinoma (ccRCC), the loss of von Hippel–Lindau (VHL) inhibition of HIF promotes glycolysis and lactate production, leading to H3K18la enrichment at the platelet-derived growth factor receptor beta (PDGFRB) promoter, increasing its transcription, forming a positive feedback loop promoting glycolysis, and promoting tumor angiogenesis [179]. In clear cell renal cell carcinoma, FK506 binding protein 10 (FKBP10) directly binds to LDHA through its C-terminus, increasing LDHA-Y10 phosphorylation and promoting glycolysis. The overactive Warburg effect promotes H3K14la, H3K18la and H3K56la, causing a series of downstream reactions that promote tumor proliferation and metastasis [180].

Studies have shown that KAT8 can regulate mitochondrial energy metabolism by modulating the lactylation of MDH2, thereby promoting the progression of renal cell carcinoma. Future research should investigate novel mitochondrial substrate proteins that are lactylated by KAT8 and their relevance to diseases [224]. A major challenge in the treatment of clear cell renal cell carcinoma (ccRCC) is the lack of ideal therapeutic targets in patients with advanced metastatic tumors or postoperative recurrence. Patients generally exhibit initial insensitivity or long-term resistance to widely used immunosuppressants, tyrosine kinase inhibitors, and novel HIF2α inhibitors. Therefore, an urgent need exists to further understand and explore efficient therapeutic targets and medication strategies based on the prominent genetic and metabolic characteristics of ccRCC [180].

#### Lactylation in prostate cancer

HIF1α lactylation increases its stability and levels, increasing cell migration-inducing protein and hyaluronan binding (KIAA1199) transcription and promoting hyaluronic acid (HA)/VEGFA signalling-mediated angiogenesis and vascular mimicry to induce tumor growth and metastasis [225]. HIF1α lactylation promotes its stable expression, increases PD-L1 transcription, and impairs sema3A transcription, weakening its inhibition of angiogenesis and promoting tumor angiogenesis and immunosuppression [226]. Gambogic acid (GBA) recruits the delactylase SIRT1 and delactylates CNPY3, affecting the cellular localization of CNPY3 and leading to tumor cell lysosomal rupture and apoptosis [133].

Studies have shown that HIF1α lactylation plays core roles in tumor angiogenesis, vasculogenic mimicry, and immune evasion by stabilizing its function and regulating downstream target genes, resulting in the formation of a “metabolism–epigenetics–vascular/immune” synergistic cancer-promoting network [225, 226]. The therapeutic potential of targeting lactylation using gambogic acid is verified through the recruitment of SIRT1 to mediate CNPY3 delactylation and induce apoptosis [133]. The androgen receptor signalling inhibitor (ARSI) enzalutamide (Enz) has shown efficacy in the treatment of advanced prostate cancer (PCa). However, the development of drug resistance is a major cause of death of prostate cancer patients; thus, more new therapeutic targets need to be identified in the future [227].

#### Lactylation in bladder cancer

circXRN2 binds to the speckle-type POZ protein (SPOP) degron, preventing SPOP-mediated degradation of large tumor suppressor kinase 1 (LATS1), a key molecule in the Hippo kinase cascade. The activation of the Hippo signalling pathway inhibits H3K18 lactylation and LCN2 expression in human bladder cancer and suppresses tumor progression [182]. Phosphofructokinase-1 (PFK1) knockdown inhibits lactylation, reduces H3K18 and ZEB1 promoter binding, decreases ZEB1 generation, inhibits the EMT, and reduces tumor metastasis [183]. Lactylated H3K18 is enriched at the promoter region of target genes, activates transcription, upregulates the key transcription factors YBX1 and YY1, and promotes cisplatin resistance in bladder cancer (BCa) [184].

Cisplatin-based combination chemotherapy has become an important adjuvant treatment for patients with BCa. Current studies have revealed the role of H3K18 lactylation in cisplatin resistance, and future research needs to further investigate the upstream and downstream molecular regulatory mechanisms to provide a basis for targeted therapy [184]. Lactylation promotes tumor metastasis by regulating transcription factors and the EMT, revealing a regulatory cascade of glycolytic metabolism–lactylation modification–EMT, which provides a basis for targeting metabolism to intervene in tumor metastasis [183]. Histone lactylation and acetylation share lysine sites and exhibit competitive binding, and the specific mechanism remains to be explored [182].

#### Lactylation in ovarian cancer

Lactate promotes H3K18la expression, polarizing macrophages to the M2 type and promoting CCL-18 expression in macrophages. CCL-18 promotes tumor growth and metastasis both in vitro and in vivo [185]. Tanshinone I reduces the expression of glycolytic enzymes such as HK2, PFK (phosphofructokinase), ENO2 (enolase 2), and LDHA, decreasing lactate production, downregulating H3K18la, reducing the expression of tumor-related genes such as TTK, PDGFRβ, YTHDF2, and RUBCNL, and inhibiting tumor growth [186].

Histone lactylation activates the homologous recombination (HR) repair pathway to promote drug resistance. For example, lactylation at the H3K9 site transcriptionally activates core genes involved in HR repair (such as RAD51 and BRCA2), thereby increasing the HR repair capacity [228]. Current research lacks sufficient evidence for clinical translation, and more patients need to be included to improve the credibility of the results [228]. In the future, analyses of the regulatory network of histone lactylation can be performed. Systematic screening of histone loci (such as H3K9la and H3K18la) and target genes regulated by lactylation in ovarian cancer should be conducted to clarify whether lactylation promotes drug resistance by regulating multiple DNA repair pathways (such as HR and NER) or oncogenic pathways (such as PI3K/AKT) [229].

#### Lactylation in head and neck cancer

Lactic acid induces high levels H3K9 lactylation, transcriptionally activates IL-11 in tumor cells, and subsequently promotes the dysfunction of CD8^+^ T cells through the JAK2/STAT3 signalling pathway, leading to the exhaustion of CD8^+^ T cells in patients with head and neck squamous cell carcinoma (HNSCC) and an unfavourable response to immunotherapy [187, 230]. The oncogene B-RAF serine/threonine protein kinase V600E (BRAFV600E) increases glycolytic flux to reorganize the cellular lactylation landscape, resulting in H4K12 lactylation-driven gene transcription and cell cycle dysregulation. H4K12la, in turn, induces the expression of multiple genes necessary for the proliferation of anaplastic thyroid carcinoma (ATC) [188]. The multifunctional RNA/DNA helicase DHX9 is involved in DNA replication, transcription, and RNA processing and inhibits cell proliferation, migration, and invasion. Abnormal expression of DHX9 can lead to DNA damage and a decrease in genomic stability [231, 232]. When DHX9 undergoes lactylation, its protective effect is reversed, and it promotes the development of oral squamous cell carcinoma (OSCC) [233]. H3K18 lactylation and H3K27 lactylation are upregulated in OSCC, promoting the expression of basal cell adhesion molecule (BCAM), which in turn promotes the invasion, angiogenesis, and chemoresistance of OSCC [189].

Studies have shown that lactylation is involved in key processes such as immune evasion, metabolic reprogramming, proliferation regulation, invasion, metastasis, and therapeutic resistance in various head and neck cancers through the targeted regulation of histones (e.g., H3K9la, H4K12la, H3K18la, and H3K27la) and nonhistones (e.g., DHX9) [187, 188, 231, 234]. Future research should investigate whether lactylation is involved in immune evasion by regulating the functions of immune cells such as tumor-associated macrophages (TAMs) and dendritic cells (DCs) to improve the “tumor cell–immune cell” lactylation regulatory network [187]. Some studies rely on cell lines, and tissue heterogeneity needs to be considered. The highly heterogeneous microenvironment in clinical tumor tissues (such as infiltrating immune cells, stromal cell crosstalk, and differences in the metabolic environment) should be simulated to more comprehensively reflect the real in vivo patterns regulating lactylation [233].

#### Lactylation in haematolymphoid system cancer

In acute myeloid leukaemia (AML), the upregulation of STAT5 promotes lactate accumulation, increases the nuclear translocation of E3BP, and facilitates histone H3K18la, ultimately inducing PD-L1 transcription and leading to immunosuppression [191]. Lactic acid upregulates H3K18la and METTL3la, leading to the accumulation of METTL3 and promoting all-trans retinoic acid (ATRA) resistance in acute promyelocytic leukaemia (APL) [190]. Mucin 20 (MUC20) inhibits the activation of the MET proto-oncogene receptor tyrosine kinase by suppressing the lactylation of insulin-like growth factor receptor-1 (IGF-1R) in multiple myeloma (MM) cells, thereby alleviating resistance to proteasome inhibitors (PIs) [235].

In acute lymphoblastic leukaemia, the competitive or synergistic relationships between histone lactylation (e.g., H4K5la) and other posttranslational modifications (such as acetylation and methylation) in the PD-L1 promoter region have not yet been studied, which hinders a comprehensive understanding of the epigenetic regulatory network [191]. Current studies have identified the key role for PD-L1 in the immune evasion of AML, but the lack of systematic clinical validation limits its clinical translational application [191]. In the future, efforts should be made to further clarify the mechanisms of action of key molecules and promote clinical translational research.

This figure summarizes validated lactylation sites on histones (e.g., H3K9la, H3K14la, H3K18la, H3K56la, H4K12la) and key non-histone lactylation events across major malignancies. Each colored cell denotes the functional outcome of the indicated lactylation mark in the corresponding cancer type: ↑ promotion, ↓ suppression, ↔ feedback regulation; these marks are further linked to tumor progression, immune evasion, metabolic reprogramming, or chemotherapy resistance.

Lactylation circuitry within the tumor microenvironment orchestrates immune evasion. Tumor-derived lactate (transported via MCT1/2/4) accumulates in the acidic milieu, triggering histone (H3K18la, H3K9la) and non-histone (APOC2 K70la, MOESIN K72la) lactylation events in malignant cells, stromal fibroblasts, and infiltrating immune cells. These lactyl marks:

- Polarize tumor-associated macrophages toward an M2 phenotype (TAM H3K18la↑ → TNFSF9, GPD2, VEGF, ARG1), suppressing IL-12 and IL-10 secretion.
- Drive myeloid-derived suppressor cell (MDSC) expansion (C163pa → lactate↑ → MDSC) and inhibit dendritic cell maturation (SREBP2^CD63^ mregDC).
- Enhance cancer-associated fibroblast (CAF) activation (GPR65-cAMP/PKA/CREB → HMGB1) and mesenchymal stem cell recruitment, fostering extracellular-matrix deposition.
- Up-regulate PD-L1 on tumor cells (MCT1/NF-κB/COX-2) and on neutrophils, while down-regulating cytotoxic molecules in NK and CD8⁺ T cells (impaired p38 MAPKs, JNK/c-Jun, NFAT → ↓IFN-γ, ↓GrB).
- Promote regulatory T-cell (Treg) proliferation (APOC2 K70la → Treg↑) and Th17 skewing (Tumor H3K9la → IL-11↑ → SMAD3).

Conversely, lactate-mediated HDAC inhibition (HDAC↓) restores Tcf7 H3K27ac, enabling CD8⁺ T-cell activation under certain contexts.

Collectively, lactylation-dependent signaling cascades—Notch/Hes1, HCAR1, GM-CSF/IL-6, and XBP1-MGAT1—coordinate a multi-cellular immunosuppressive network that supports tumor progression, angiogenesis, and therapeutic resistance.

## Structures of lactate and lactylation-related proteins

**Fig. 4:**
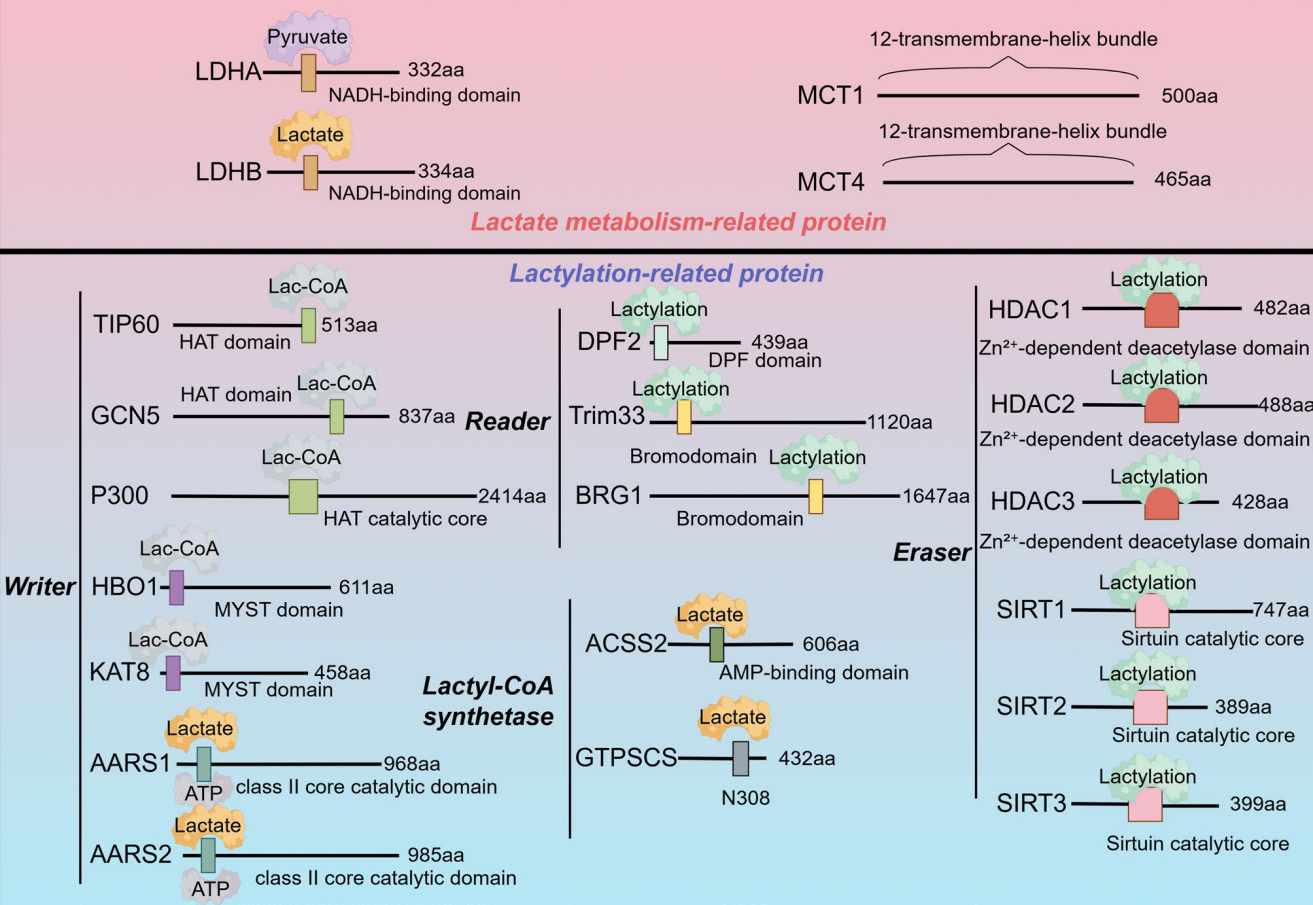
The structure of lactate and lactylation-related proteins

Here, we summarize the structures of lactate and lactylation-related proteins, which hold significant reference value for targeted therapy (Fig. 4) (Table 4).

**Table 4:** The structure of lactate and lactylation-related proteins

Protein	Key structural domain/site	Recognition/action target	Reference
LDHA	NADH-binding domain	Pyruvate	[236]
LDHB	NADH-binding domain	Lactate	[237]
MCT1	12-TM bundle	Lactate	[238]
MCT4	12-TM bundle	Lactate	[238]
ACSS2	AMP-binding domain (D358)	Lactate	[115]
GTPSCS	N308	Lactate	[114]
P300	HAT catalytic core	Lactyl-CoA	[113]
GCN5	HAT domain	Lactyl-CoA	[115]
TIP60	HAT domain	Lactyl-CoA	[144]
HBO1	MYST domain	Lactyl-CoA	[128]
KAT8	MYST domain	Lactyl-CoA	[124]
AARS1/2	Class II core catalytic domain	Lactyl-AMP	[123, 239]
DPF2	DPF domain	Lactylation	[137]
TRIM33	Bromodomain	Lactylation	[138]
BRG1	Bromodomain	Lactylation	[139]
HDAC1-3	Zn^2^⁺-dependent deacetylase domain	Lactylation	[129]
SIRT1-3	Sirtuin catalytic core	Lactylation	[129, 157]

LDHA is encoded by the LDHA gene and typically exists as a tetramer (LDH-5) [236]. It consists of 332 amino acids, with residues 99–110 forming a flexible “active site loop” conformation, commonly referred to as the substrate-specific loop, which facilitates LDHA catalysis [240]. Among these amino acids, arginine 109 is believed to stabilize the transition state during hydride transfer reactions [241]. Residues 20–162 and 248–266 form a large Rossmann domain characterized by three parallel β-strands surrounding two α-helices, which serves as the cofactor binding site. At this site, residues such as Asp168, ARG171, Thr246, and the catalytic His195 play crucial roles in binding to the NADH cofactor [242–244], contributing to the catalytic activity of LDHA. Residues 163–247 and 267–331 form a mixed α/β substrate-binding domain. Substrates, such as pyruvate, primarily interact with residues such as ARG171, Thr246, and Ala236 [245]. The active site loop, cofactor binding site, and substrate binding site form a specific spatial conformation that collectively promotes LDHA catalysis. Therefore, these sites represent ideal targets for inhibitor action.

LDHB is encoded by the LDHB gene and is expressed mainly as the LDH1 (H4) isoform, which is abundant in cardiac and brain tissues and preferentially catalyses the oxidation of lactate to pyruvate [246]. LDHA and LDHB share considerable sequence and structural homology, but their subcellular localization and substrate affinity differ, leading to distinct functional roles [247]. Current studies have identified His178 as the catalytic residue responsible for LDHB function [248].

Researchers have predicted that all members of the SLC16 family share a topological structure featuring 12 transmembrane helices (TMs) with intracellular C-terminal and N-terminal regions, as well as a large cytoplasmic loop between TMs 6 and 7. This family is defined by two highly conserved sequences, [D/E]G[G/S][W/F][G/A]W and YFxK[R/K][R/L]xLAx[G/A]xAxAG, which traverse TM1 and TM5, respectively [249]. The human MCT1 model identifies lysine 38, aspartate 302, and arginine 306 as particularly important for its substrate binding and transporter activity [250]. Compared with MCT1, which is more commonly found in cells with active oxidative metabolism, MCT4 is more prevalent in highly glycolytic cells. It has the lowest affinity for lactate among MCTs and primarily facilitates lactate efflux from glycolytic cells [29].

ACSS2 functions as a lactate coenzyme A synthetase, converting lactate to lactyl-CoA. ACSS2 adopts two conformations: closed and open. Acetate and lactate form hydrogen bonds with T363 of ACSS2 in the closed state, whereas in the open state, acetate forms hydrogen bonds with Y315 and R202 of ACSS2, respectively, and lactate forms a hydrogen bond with D358 of ACSS2 [115].

GTPSCS also functions as a lactyl-CoA synthetase. The C-terminal region of the G2 subunit of the GTPSCS is responsible for substrate (succinate or lactate) binding. Lactate is embedded in a hydrophobic surface formed by residues of the G2 and G1 subunits. The lactate carboxylate group interacts with the side chain of N308 via hydrogen bonds and is further stabilized by three hydrogen bonds between its hydroxyl group and the nitrogen atoms of G365/V367/N308. The N308I mutation almost completely abolishes lactyl-CoA synthetase activity in vitro, indicating its critical role [114].

p300 plays important roles in integrating multiple signal transduction pathways in the nucleus and mediating enhancer-driven transcription [251]. p300 has an ordered catalytic “core” domain, including a histone acetyltransferase (HAT) domain and bromodomain (BD), as well as several other domains that form promiscuous interactions with the disordered activation domains (ADs) of hundreds of cellular transcription factors (TFs) [252]. These interaction domains are separated by long, intrinsically disordered regions (IDRs). The p300 core contains RING and TAZ2 domains with autoinhibitory functions; mutations in these domains disrupt autoinhibition, leading to constitutive p300 activation [253].

Numerous studies have shown that GCN5 (and the related P/CAF) is a conserved HAT, and its activity on nucleosomes helps initiate transcription [254]. Its domain includes residues 99–262 of the 439-aa protein [255]. The Gcn5-related N-acetyltransferase (GNAT) superfamily is a large protein superfamily that exhibits a common fold consisting of 6–7 antiparallel β-strands and 4 α-helices in the β1-α1-α2-β2-β3-β4-α3-β5-α4-β6-β7 topology. The amino acid sequence of the Gcn5 HAT contains four conserved motifs, A–D, arranged in the order C-D-A-B, where motif A contains a P-loop that binds CoA pyrophosphate. Motifs A and B form the substrate-binding cleft, and a β-bulge in strand β4 provides an oxyanion hole important for stabilizing the tetrahedral intermediate [256].

Mammalian TIP60 is a multifunctional enzyme with histone acetylation and histone dimer exchange activities. TIP60 is a large complex composed of 17 subunits, including MBTD1, E1A binding protein p400 (EP400), DMAP1, YEATS domain containing 4 (YEATS4), ACTL6A, ACTB, EPC1, KAT5, ING3, MEAF6, MRG15, MRGBP, TRRAP, YL1, RUVBL1, RUVBL2, and BRD825 [257, 258]. TIP60 can be divided into five modules, TINTIN, HAT, TRRAP, ARP, and BASE, each with distinct functions. Within the complex, the EP400 protein acts as the main scaffold, interacting with all functional and structural modules. Following the unstructured N-terminal domain of EP400 and preceding its motor domain, the prehuman serum albumin (HSA) region and part of the HSA region interact with DMAP1, ACTB, and one BAF53a subunit to form the ARP module. The ATP-dependent motor domain, EP400 insertion domain, and hexameric AAA-ATPase constitute most of the BASE module below the ARP module. The postmotor domain (PMD) of EP400 folds towards the N-terminus, integrating itself into the ARP module and contributing to the surface that connects the HAT module to the rest of the complex. After a long, disordered linker, the SANT and HD domains of EP400 directly interact with the TRRAP module, flexibly linking it to the rest of TIP60 [259].

The MYST family contains the greatest number of HAT proteins in humans, including MOF (males-absent-on-the-first), KAT8, and MYST1 [260]. The HBO1 complex is the major acetyltransferase responsible for histone H4 acetylation in vivo and belongs to the MYST family [261]. As the core catalytic subunit, HBO1 consists of an N-terminal domain responsible for acetyl-CoA binding and acetylation reactions [262] and a C-terminal MYST domain [263]. The HBO1 complex is multimeric and is typically composed of two native subunits (MEAF6, ING4, or ING5) and two accessory factors that act as chromatin readers: Jade-1/2/3 and BRPF1/2/3 [264, 265]. HBO1 functions as a lysine lactyltransferase to regulate transcription; E508 is a key site for HBO1 lactyltransferase activity, and scaffold proteins, including jumonji and AT-rich interactive domain 1 (JADE1) and BRPF2 can promote its histone Kla enzymatic activity [128]. The chromodomain (Δ1 to 121 aa) and C2HC zinc finger (Δ121 to 232 aa) of KAT8 are dispensable for global protein lactylation, whereas the enzymatic MYST domain is essential for its lactyltransferase activity [124].

AARS1/2 act as lactate sensors, mediating global lysine lactylation in tumor cells. Lactate is initially activated by AARS in an ATP-dependent manner via the reaction intermediate lactyl-AMP, which is then transferred and conjugated to lysine residues of target proteins. Lactate molecules are docked into the crystal structure of human AARS1/2 [239]. Lactate molecules overlap with the alanine moiety of Ala-SA (an Ala-AMP analogue), suggesting that lactate may overlap with the binding site of L-alanine when it interacts with human AARS1/2 [123].

DPF2 functions as a reader for histone lactylation. DPF2 recognizes histone lactylation sites by adopting a binding model similar to that of histone acetylation, where the N-terminal α-helix of H3 engages with the second PHD finger (PHD2) through electrostatic and hydrogen bond interactions (e.g., D346 and L342), while Kla is anchored in the hydrophobic pocket of the first PHD finger (PHD1), involving key residues such as D274, F275, R300, and E326. Notably, the hydroxyl group within Kla interacts with the residue D274 via two direct hydrogen bonds, increasing the recognition of H3K14la by DPF2 [137].

TRIM33 is a member of the tripartite motif (TRIM) protein family and is characterized by a typical N-terminal tripartite motif containing a RING finger domain, one or two zinc finger domains (B1 box and B2 box), and an associated coiled-coil region [266]. A unique glutamic acid residue within the TRIM33 binding pocket confers specificity for Kla binding [138].

Studies by Hu et al. have shown that Brg1 can act as a lactylation reader to increase nucleosome accessibility and transcriptional activity. In this study, the model incorporated methionine at the first translation initiation site, resulting in a lysine at position 19. The unmodified H3 protein establishes nine distinct sets of hydrogen bond interactions with the N-terminal region of the Brg1 protein, whereas binding of K19 in modified H3 protein to Brg1 leads to the formation of 13 distinct sets of hydrogen bond interactions at the same binding site. The increased number of hydrogen bonds indicates that compared with unmodified H3, Brg1 has greater affinity for H3K18la-modified histone variants, suggesting its potential role as a histone lactylation “reader”. Specifically, Brg1 acts as a lactylation “reader” by binding to lactylated lysine residues through its bromodomain [139].

HDAC1–3 are class Ⅰ HDACs, with a catalytic site topology characterized by a zinc cofactor chelated by two aspartate residues and one histidine residue and a substrate binding site located at the bottom of a narrow lipophilic cleft forming the catalytic site [267]. They are zinc-dependent classical HDACs, meaning that they require a Zn^2^⁺ ion in their catalytic site for activity [268].

SIRT1–3 are class III HDACs and require NAD⁺ as a cofactor for enzymatic activity. Their catalytic domain is located in a cleft formed between a large domain with a Rossmann fold and a small zinc-binding domain. Amino acid residues in the cleft are conserved across the deacetylase family and form a protein tunnel for substrate and NAD⁺ interactions [269].

Architectural overview of the human “lactylation toolkit.” The diagram collates key proteins that produce the lactyl-donor lactyl-CoA (ACSS2, GTPSCS, AARS1/2), transport lactate (MCT1, MCT4), interconvert lactate and pyruvate (LDHA, LDHB), and catalyze the writing (lysine acetyl-transferases with HAT/MYST domains: P300, GCN5, TIP60, KAT8, HBO1), reading (DPF2, TRIM33, BRG1 bromodomains), and erasing (HDAC1-3, SIRT1-3) of lactyl marks. Proteins are annotated with their signature domains (e.g., 12-transmembrane-helix bundle, NADH-binding domain, Zn^2^⁺-dependent deacetylase, sirtuin catalytic core).

## Therapies targeting the lactylation process

Targeting lactylation is no longer confined to a single node; instead, it encompasses at least four mechanistically distinct routes—(i) upstream lactate production, (ii) membrane lactate transport, (iii) an intracellular lactyl-CoA supply, and (iv) chromatin-level “writing/erasing” of lactylation—any of which can be pharmacologically intercepted [113]. For lactate production, inhibitors of specific isoforms of pyruvate kinase, including PKM2 inhibitors such as the natural flavonoid apigenin [270, 271] and heterocyclic molecules with Se-N bonds such as isoselenazolium salts [272], have been developed. 2-Cyano-3-(1-phenyl-1H-indol-3-yl) acrylic acid (UK5099) and POx not only trigger pyroptosis through a cascade of biocatalysis to increase the immunogenicity of tumor cells but also reshape the immunosuppressive TME by targeting pyruvate metabolism [273, 274]. Additionally, LDHA inhibitors such as oxamate competitively inhibit the LDHA substrate pyruvate [275] and gossypol is a competitive inhibitor of the cofactor NADH [276, 277]. After RAW 264.7 macrophages are treated with the specific LDHA inhibitor FX11, the cellular lactate level decreases, the LPS-induced expression of IL-6, inducible nitric oxide synthase (iNOS), and COX-2 decreases, and the inflammatory response is inhibited [278]. After treatment with GNE-140, LDHA expression is inhibited, and cancer cell death occurs only two days later [279]. The selenobenzene compound 1-(phenylselanyl)−4- (trifluoromethyl) benzene (PSTMB) inhibits the function of LDHA, reduces glycolysis in cancer cells, and can inhibit the growth of various cancer cells, such as human large cell lung cancer, human breast cancer, human hepatocellular carcinoma, human malignant melanoma and human colon cancer [280]. Some studies have shown that catechins can reduce chemoresistance to 5FU in cancer cells by restricting LDHA [281]. In prostate cancer, Compound 3D downregulates the expression of lactate-regulating genes (LDHA, MCT1/4, and CAIX) and the zinc influx transporter (ZIP1) to inhibit aerobic glycolysis, resulting in selective antiproliferative activity [282]. LDH proteolytic Compound 22 (MS6105), a targeted protein degradation chimaera (PROTAC), can effectively degrade LDHA in a time- and ubiquitin‒proteasome system-dependent manner [283]. GLUT1 inhibitors include resveratrol (3,5,4′-trihydroxystilbene or RSV) [284], cytochalasin B [285, 286] and tyrosine kinase inhibitors [287]. Among these, BAY876 is a highly specific GLUT1 inhibitor that effectively suppresses the proliferation of OSCC cells by targeting GLUT1 [288].

For lactate transport, α-cyano-4-hydroxycinnamic acid (ACCA) [289] and phloretin and quercetin [290–292] target MCTs. Diclofenac also inhibits MCTs [293]. The MCT inhibitor clathrin heavy chain (CHC) reduces tumor glycolytic metabolism, migration, and invasion and induces the death of highly glycolytic cells [294, 295]. The MCT1 and MCT4 inhibitor syrosingopine disrupts cellular pH homeostasis and has a negative effect on tumor growth [296]. 7-Aminocarboxycoumarin (7ACC) selectively interferes with lactate flux in the lactate-rich tumor microenvironment, inhibits lactate influx in tumor cells expressing the MCT1 and MCT4 transporters, and delays tumor growth [297]. Specific inhibitors of MCT1 include p-chloromercuribenzenesulfonic acid (pCMBS) [298], 3-bromopyruvate (3-BrPA) [299] and AZD3965 [300]. AZD3965 is currently undergoing preclinical research. As a MCT-1 inhibitor, it can inhibit the transport of lactate out of or into cells that do not express MCT-4. Therefore, it has the potential to exploit the dependence of cancer cells on aerobic glycolysis, leading to intracellular lactate accumulation, feedback inhibition of glycolysis, and a pH imbalance. A male patient with metastatic melanoma developed refractory hyperlactatemic acidosis after taking AZD3965. The researchers speculated that a single dose temporarily interferes with plasma clearance in the liver and other organs, resulting in the deterioration of symptoms. This finding provides guidance for subsequent drug development [301]. The MCT1 inhibitor BAY-8002 effectively inhibits bidirectional lactate transport, which can arrest tumor development [302]. The MCT1 inhibitor AR-C155858 inhibits the uptake of L-lactate in a time-dependent manner, and a certain amount of time is required to fully reverse its effects after removal, indicating slow reversibility [303]. The selective inhibitors of MCT4 include carboxycoumarin derivatives [304] and 2-((5-(3-cyclopropoxyphenyl)−1-phenyl-1H-pyrazol-3-yl) methoxy)−2-methylpropanoic acid (VB124) [238, 305]. Additionally, the MCT4 inhibitor AR-C122982 overcomes the radiation resistance of tumor cell lines [295]. Acridine flavine (ACF) inhibits the binding between basigin and MCT4, reduces the functional plasma membrane expression of MCT4, and ultimately inhibits angiogenesis and tumor progression [306].

With respect to lactate itself, lactate oxidase (LOX) specifically catalyses the oxidation of lactate to pyruvate, resulting in the significant consumption of lactate [307]. Acid-neutralizing calcium carbonate nanoparticles (nanoCaCO3) buffer the pH within the normal physiological range and inhibit tumor cell proliferation [308]. 3-Hydroxybutyrate (3-OBA) inhibits lactate production, reverses the effects of hypoxia, such as the M2 polarization of macrophages, and inhibits the growth and metastasis of CRC cells [309].

With respect to the lactate receptor, gallein, an inhibitor of the Gβγ subunit of G protein-coupled receptors (GPCRs), can inhibit the migration of TGF-α-induced HuH7 HCC cells [310].

With respect to lactyl-CoA synthetase, combined treatment with a peptide that blocks the ACSS2–KAT2A interaction and an anti-PD-1 antibody can interfere with Warburg effect-induced and lactate-dependent chromatin modifications, as well as tumor immune evasion and growth [115].

Treatments targeting lactylation "writers" include inhibitors such as anacardic acid from cashew nut shells and gambogic acid from Garcinia, which inhibit p300/CBP [311, 312]; competitive p300 inhibitors such as C646; catalytic inhibitors of p300 and CBP such as A-485 [313, 314]; and L-alanine, which inhibits AARS1 [122].

Relevant therapies targeting lactylation "erasers" have also been developed. Tucidinostat inhibits HDAC2 expression, thereby suppressing the delactylation of METTL3 and increasing the sensitivity of triple-negative breast cancer (TNBC) cells to cisplatin treatment [220]. AGK2 inhibits SIRT2, promotes the lactylation of METTL16, enhances the therapeutic effect of the copper ionophore elesclomol, and induces cuproptosis in gastric tumors [120]. Honokiol activates SIRT3, downregulates the lactylation level of CCNE2, induces apoptosis in hepatocellular carcinoma (HCC) cells, and prevents the growth of HCC in vivo [157]. Gambogic acid (GBA) recruits SIRT1, leading to the delactylation of CNPY3 and the subsequent rupture of lysosomes to trigger pyroptosis in prostate cancer [133]. Combined treatment with a low dose of the SIRT1-specific activator SRT2104 and the LDHA inhibitor oxalate exerts a significant antitumor effect on gastric cancer (GC) cells [315].

**Fig. 5:**
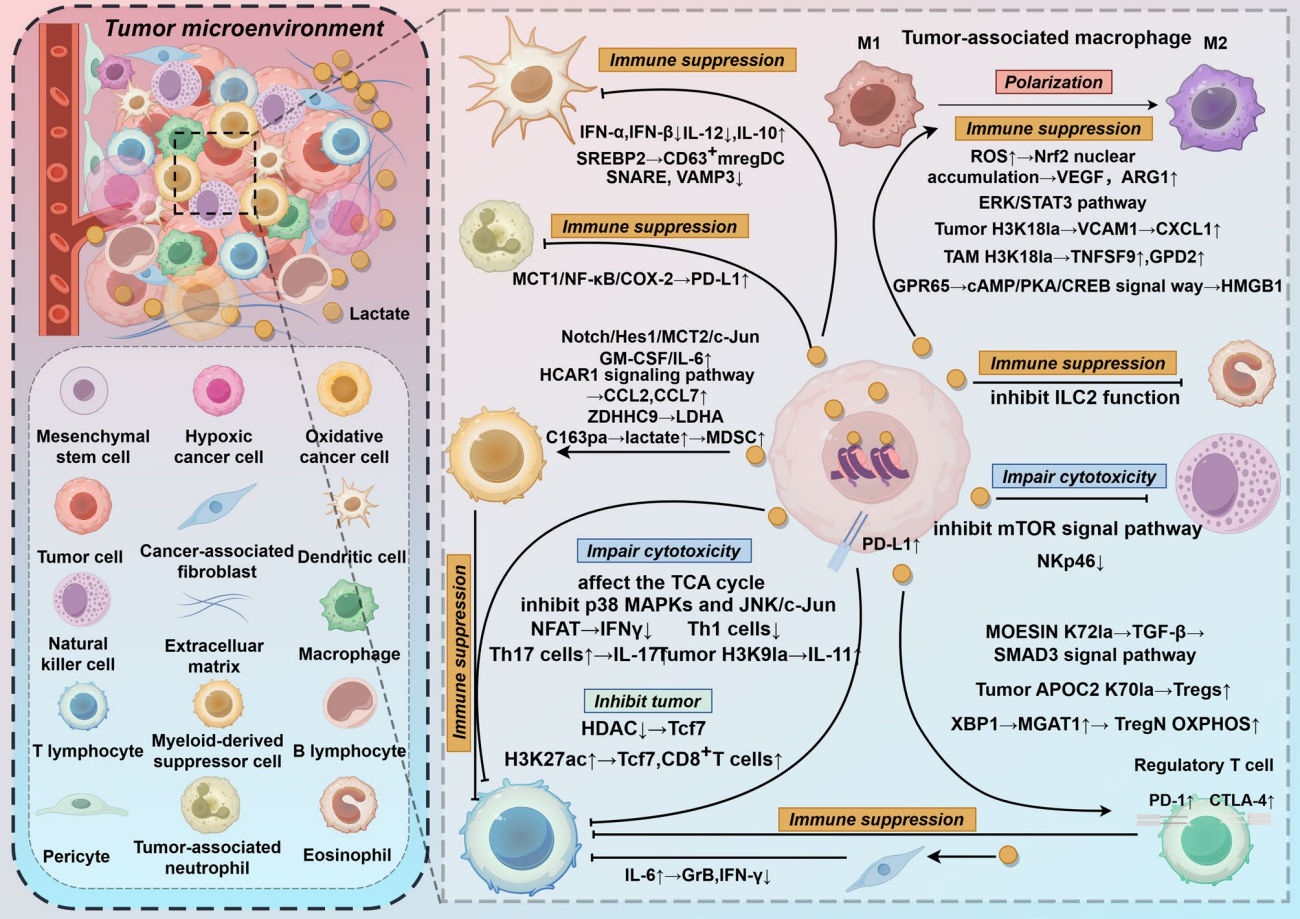
Lactate and lactylation in TME

With respect to lactylation sites, demethylzerumbone (DML) inhibits H3K9la and H3K56la, thus inhibiting tumorigenesis [316]. Royal jelly acid (RJA) inhibits the lactylation of H3 histone at the H3K9la and H3K14la sites to inhibit the development of HCC [317]. A cell-penetrating peptide (CPP) targets the lactylation of MRE11 K673, impairs homologous recombination (HR), and thus promotes the sensitivity of cancer cells to cisplatin and PARPis [118]. Treatment with D34-919 significantly reduces the level of X-ray repair cross complementing 1 (XRCC1) lactylation at K247 and decreases the nuclear import of XRCC1, thereby inhibiting DNA repair [169]. β-Alanine disrupts the binding of lactate to AARS1, reduces the lactylation of lysine 120 and lysine 139 in the DNA binding domain of p53, and inhibits tumorigenesis [123] (Fig. 5) (Table 5).

**Table 5:** Therapies targeting the lactylation process

Target		Drug	Application	Effect	Reference
Inhibit lactate production	GLUT	RSV	prostate cancer,breast cancer	suppress GLUT1	[284]
		cytochalasin B	CRC, renal cancer,lung cancer,breast cancer	suppress GLUT1	[285, 286]
		tyrosine-kinase inhibitors	AML	suppress GLUT1	[287]
		BAY876	OSCC	suppress GLUT1	[288]
	PK	apigenin	malignant neuroblastoma	suppress PKM2	[270, 271]
		isoselenazolium salts	breast cancer,lung adenocarcinoma	suppress PKM2	[272]
		UK5099.POx	breast cancer	suppress PK	[273]
	LDH	oxamate	medulloblastoma	suppress LDHA	[275]
		gossypol	gastric cancer	suppress LDHA	[276, 277]
		FX11	neuroblastoma	suppress LDHA	[278]
		GNE-140	pancreatic cancer	suppress LDHA	[279]
		PSTMB	CRC,lung cancer,BCa,melanoma	suppress LDHA	[280]
		catechin	gastric cancer	suppress LDHA	[281]
		3D	prostate cancer	suppress LDHA	[282]
		MS6105	pancreatic cancer	suppress LDHA	[283]
Inhibit lactate transport	MCT	ACCA	malignant neuroblastoma	suppress MCTS	[289]
		phloretin	lung cancer,CRC	suppress MCTS	[290–292]
		quercetin	lung cancer,CRC	suppress MCTS	[290–292]
		diclofenac	melanoma	suppress MCTS	[293]
		CHC	glioma,OSCC,breast cancer	suppress MCTS	[294, 295]
		syrosingopine	Breast cancer and pharyngeal squamous cell carcinoma	suppress MCTS	[296]
		7ACCs	Cervix cancer,CRC,Breast cancer	suppress MCTS	[297]
		pCMBS	CRC	suppress MCT1	[298]
		3-BrPA	cervical cancer,bladder cancer	suppress MCT1	[299]
		AZD3965	small cell lung cancer	suppress MCT1	[300]
		BAY-8002	diffuse large B-cell lymphoma,solid tumor	suppress MCT1	[302]
		AR-C155858	breast cancer	suppress MCT1	[303]
		carboxycoumarin derivatives	cervical cancer	suppress MCT4	[304]
		VB124	HCC	suppress MCT4	[305, 318]
		AR-C122982	OSCC	suppress MCT4	[295]
		ACF	glioblastoma	suppress MCT4	[306]
Regulate PH	lactate	LOX	CRC,breast cancer, melanoma	suppress lactate	[307]
		nanoCaCO3	breast cancer	suppress lactate	[308]
		3-OBA	CRC	suppress lactate	[309]
Inhibit GPR	GPR	gallein	HCC, prostate cancer	suppress GPR	[310]
Inhibit lactyl-CoA synthetase	ACSS2	ACSS2-KAT2A interaction blocking peptide	GBM	suppress ACSS2	[115]
Inhibit writers	p300/CBP	anacardic acid	cervical cancer	suppress p300/CBP	[311]
	p300/CBP	gambogic acid	cervical cancer	suppress p300/CBP	[312]
	p300	C646	pancreatic cancer	suppress p300	[313, 314]
	p300/CBP	A-485	prostate cancer	suppress p300/CBP	[313]
	AARS	L-alanine	gastric cancer	suppress AARS1	[122]
Regulate erasers	HDAC	Tucidinostat	TNBC	suppress HDAC2	[220]
	SIRT	AGK2	gastric cancer	suppress SIRT2	[120]
		Honokiol	HCC	activate SIRT3	[157]
		GBA	prostate cancer	recruit SIRT1	[133]
		SRT2104	gastric cancer	activate SIRT1	[315]
Inhibit lactylation	histone lactylation	DML	HCC	suppress H3K9la and H3K56la	[316]
		RJA	HCC	suppress H3K9la and H3K14la	[317]
	nonhistone lactylation	CPP	CRC	suppress MRE11 K673la	[118]
		D34-919	glioblastoma	suppress XRCC1 K247la	[169]
		β-alanine	CRC	Suppress p53 K120/139la	[123]

## Conclusions and perspectives

This review summarizes the metabolism and functions of lactic acid, lactylation, the structures of lactate and lactylation-related proteins and therapeutic drugs targeting the lactylation process, with a focus on elaborating on the important roles of lactic acid and lactic acid-driven lactylation in the occurrence and development of cancer.

The identification of histone lactylation has established a novel mechanistic link between cellular metabolic flux and epigenetic reprogramming, positioning this posttranslational modification as an emerging frontier in oncology research. While substantial progress has been made in characterizing lactylation-associated pathways in recent years, critical gaps persist in mapping the complete regulatory network of this modification. Notably, the enzymatic machinery governing lactylation dynamics—including specific “writers” that catalyse lysine lactylation, “readers” that recognize lactylate residues, and “erasers” that mediate delactylation—remains insufficiently characterized. Current studies have revealed that these enzymes exhibit significant differences in their specific expression patterns and functions across various cancer types, providing a new perspective for deciphering tumor heterogeneity. Writers and erasers of lactylation are tissue specific, and the expression levels and activities of different enzymes vary significantly among different cancers. For instance, in prostate cancer, TIP60 acts as a specific writer for the NBS1 protein, promoting DNA repair and chemoresistance by catalysing lactylation at the K388 site [144]. In pancreatic cancer, however, high expression of the histone acetyltransferase p300 leads to elevated lactylation levels at the H3K18 site, activating the transcription of nerve invasion-related genes [116]. HDAC3 specifically erases the lactylation of NBS1 in prostate cancer [144], whereas HDAC2 specifically eliminates the lactylation of METTL3 in triple-negative breast cancer, resulting in drug resistance in tumors [221]. The regulatory role of the tumor microenvironment also contributes to differences in lactylation writers and erasers among various cancers.

Different cancers exhibit varying sensitivities to lactate. In breast cancer, for example, a high-lactate environment increases FOXP3 lactylation by the p300–CBP complex, promoting Treg cell function [319]. In gliomas, the EGFR–ERK pathway activates the ACSS2/p300 complex, leading to the local production of lactyl-CoA and specifically driving histone H3K18 lactylation [320]. The activity of lactyltransferases is also dynamically regulated by cancer-specific signalling pathways. Under hypoxic conditions, HIF-1α upregulates the expression of glycolytic genes and promotes p300-mediated lactylation, such as the activation of HK2 in HCC [319]. In gastric cancer, AARS1 forms a positive feedback loop by lactylating the YAP–TEAD complex, driving tumor cell proliferation [321].

Furthermore, a critical gap in the temporal dimension remains to be addressed: in the tumor microenvironment, does lactylation first occur in cancer cells, stromal cells, or immune cells? Clarifying this sequence may redefine the causal chain of metabolic–immune reprogramming during tumorigenesis. The existing data point to a temporal sequence: “Tumor cells first produce large amounts of acid → the local lactic acid concentration rises sharply → tumor cells themselves undergo lactylation first → immune cells and stromal cells are subsequently lactylated in the acidified environment". However, due to the lack of dynamic tracking studies, the possibility that individual immune cells or stromal cells undergo lactylation at an extremely early stage cannot be excluded [322, 323]. Intriguingly, preliminary evidence suggests significant functional overlap between lactylation-related enzymes and the well-characterized acetylation/deacetylation system, suggesting potential cross-regulatory mechanisms among distinct acylation events that warrant systematic investigation [324]. Current research has revealed that a complex regulatory network involving lactylation, acetylation, and ubiquitination is involved. First, competition for occupancy has been observed among three proteins: p53 K120/K139, histones H3K18/H3K27, and PKM2-K305 can all be modified by Kla (lysine lactylation), Kac (lysine acetylation), and Ub (ubiquitination). Once Kla is formed, it prevents further recognition by the acetyltransferase p300 or the E3 ligase mouse double minute 2 (MDM2) [123]. Second, the three modifications mutually regulate the activity of related enzymes: AARS1, a Kla “writer”, competes with p300 for lactyl-CoA; HDAC3 and SIRT2 possess both deacetylase and delactylase activities, and elevated lactyl-CoA levels inhibit their delactylation efficiency, thereby relatively preserving Kac [129]. They also exhibit functional regulation; for example, in liver cancer, acetylation of PDHX at K488 (PDHX-K488ac) hinders the interaction between PDHX and dihydrolipoyl transacetylase (E2), thereby disrupting PDC assembly to inhibit its activation. This process leads to the conversion of most glucose into lactate, which facilitates aerobic glycolysis and H3K56 lactylation-mediated gene expression, promoting tumor progression [156]. Lactylation of TFEB at K91 prevents the interaction between TFEB and the E3 ubiquitin ligase WWP2, thereby inhibiting TFEB ubiquitination and proteasomal degradation, which in turn increases TFEB activity and autophagic flux [152]. However, research on the interactions between lactylation and other posttranslational modifications (PTMs) remains limited and requires further investigation.

From a translational perspective, accumulating data implicate lactylation in multiple hallmarks of cancer progression [325–327]. However, the functional conservation versus context-dependent variability of lactylation-mediated pathways across heterogeneous tumor types requires rigorous investigation. Methodological advances in three key areas could propel this field forwards: 1) the development of site-specific lactylation detection tools, 2) functional characterization of lactylation regulatory enzymes using CRISPR-based screening platforms, and 3) pharmacological modulation of lactylation flux in preclinical models. Elucidating the molecular architecture of lactylation signalling networks may reveal novel therapeutic vulnerabilities, potentially expanding the current arsenal of targeted anticancer strategies.

## Data Availability

No datasets were generated or analysed during the current study.

## Abbreviations

3-BrPA: 3-Bromopyruvate
5FU: 5-Fluorouracil
7ACC: 7-Aminocarboxycoumarin
AARS: Aminoacyl-tRNA synthetase
ABC: ATP-binding cassette
ABCF1: ATP binding cassette subfamily F member 1
ACCA: α-Cyano-4-hydroxycinnamic acid
ACF: Acridine Flavine
ACSS2: Acetyl-CoA synthetase 2
AD: Activation domains
AICD: Activation-induced cell death
AKR1B10: Aldo-keto reductase family 1 member B10
AKT/AkT: Protein kinase B
ALDOA: Aldolase A, Fructose-Bisphosphate
ALDOB: Aldolase B
ALKBH3: Alpha-ketoglutarate-dependent dioxygenase homolog 3
AML: Acute myeloid leukemia
APL: Acute promyelocytic leukemia
APOC2: Apolipoprotein C2
ARG1: Arginase-1
ASCT2: Alanine, Serine, Cysteine Transporter 2
ATF4: Activating transcription factor 4
ATC: Anaplastic thyroid carcinoma
ATRA: All-trans retinoic acid
BCa: Bladder cancer
BCAM: Basal cell adhesion molecule
Bcl-x: B-cell lymphoma-extra large
BECN1: Beclin 1
BNIP3: BCL2/adenovirus E1B 19 kDa interacting protein 3
BRAFV600E: V600E mutation in B-Raf proto-oncogene serine/threonine-protein kinase
Brg1: Brahma-related gene 1
BUB1B: BUB1 mitotic checkpoint serine/threonine kinase B
BZW2: Basic leucine zipper and W2 domains 2
CAFs: Cancer-associated fibroblasts
CBP: CREB-binding protein
CBX3: Chromobox homolog 3
CCL-18: C-C motif chemokine ligand 18
CCND1: Cyclin D1
CCNE2: Cyclin E2
CCNB1: Cyclin B1
CDK7: Cyclin-dependent kinase 7
CEACAM6: CEA cell adhesion molecule 6
CENPA: Centromere protein A
CHC: Clathrin heavy chain
circATXN7: CircRNA ataxin 7
circXRN2: Circular RNA XRN2
CNPY3: Canopy FGF signaling regulator 3 / canopy 3 homolog
CoA: Coenzyme A
COX-2: Cyclooxygenase-2
CPP: Cell-penetrating peptide
CRC: Colorectal cancer
CRM1: Chromosome region maintenance 1
CTHRC1: Collagen triple helix repeat containing 1
CTL: Cytotoxic T lymphocyte
CXCL8: C-X-C motif chemokine ligand 8 / interleukin-8
DBD: DNA-binding domain
DCBLD: Discoidin, CUB and LCCL domain-containing protein
DCs: Dendritic cells
DDX17: DEAD-box helicase 17
DLAT: Dihydrolipoamide S-acetyltransferase
DML: Demethylzerumbone
DPF2: Double PHD fingers 2
E3BP: Pyruvate dehydrogenase complex component X
eEF1A2: Eukaryotic translation elongation factor 1 alpha 2
EGFR: Epidermal growth factor receptor
EMT: Epithelial-mesenchymal transition
ENO1: Enolase 1
ENO2: Enolase 2
EOFF1A2: Eukaryotic translation elongation factor 1 alpha 2
ERK: Extracellular signal-regulated kinase
ERRα: Estrogen-related receptor alpha
ERS: Endoplasmic reticulum stress
FDX1: Ferredoxin 1
FGS: Fargesin
FKBP10: FK506 binding protein 10
FOXP3: Forkhead box P3
G6PD: Glucose-6-phosphate dehydrogenase
GBA: Gambogic acid
GBM: Glioblastoma
GC: Gastric cancer
GCN5: General control non-repressed 5
GDF15: Growth differentiation factor 15
GDH: Glutamate dehydrogenase
GCLC: Glutamate-cysteine ligase catalytic subunit
GLUT: Glucose Transporter
GLUT1: Glucose transporter 1
GLUT3: Glucose transporter 3
GM-CSF: Granulocyte-macrophage colony-stimulating factor
GNAT13: Gcn5-related N-acetyltransferase 13
GPD2: Glycerol-3-phosphate dehydrogenase 2
GPCRs: G protein-coupled receptors
GPR81: G-protein-coupled receptor 81
GPR132: G-protein-coupled receptor 132
GP73: Golgi transmembrane glycoprotein 73
GRP78: Glucose-regulated protein 78
GSCs: Glioma stem cells
GTPSCS: GTP-specific succinyl-CoA synthetase
HCC: Hepatocellular carcinoma
HDAC: Histone deacetylase
Hes1: Hairy and enhancer of split-1
HIF-1: Hypoxia-inducible factor-1
HK2: Hexokinase 2
HMGB1: High mobility group box 1
HNRNPF: Heterogeneous nuclear ribonucleoprotein F
HPV16 E6: Human papillomavirus type 16 E6 protein
HR: Homologous recombination
HAS: Human serum albumin
ICC/iCCA: Intrahepatic cholangiocarcinoma
IDH3G: Isocitrate dehydrogenase 3 gamma
IFN-γ: Interferon-gamma
IGF1: Insulin-like growth factor 1
IL: Interleukin
IL-17: Interleukin-17
iNOS: Inducible Nitric Oxide Synthase
iPSC: Induced pluripotent stem cell
IRS1: Insulin receptor substrate 1
JADE1: Jumonji and AT rich interactive domain 1
JNK: C-jun n-terminal kinase
KAT2A: Lysine acetyltransferase 2A
KCNK1: Potassium two pore domain channel subfamily K member 1
KDM3A: Lysine-specific demethylase 3A
KIAA1199: Cell migration-inducing protein, hyaluronan binding
KLF15: Kruppel-like factor 15
LAMC2: Laminin subunit gamma-2
LATS1: Large tumor suppressor kinase 1
LCN2: Lipocalin 2
LINC00152: Long intergenic non-protein coding RNA 152
LLPS: Liquid-liquid phase separation
LOX: Lysyl oxidase or lactate oxidase (context-dependent)
LPS: Lipopolysaccharide
LTBP1: Latent transforming growth factor beta-binding protein 1
LUAD: Lung adenocarcinoma
MACC1: MET transcriptional regulator MACC1
MADD: MAP-kinase activating death domain-containing protein
MAPKs: Mitogen-activated protein kinases
MAPK6P4: mitogen-activated protein kinase 6 pseudogene 4
MCT: Monocarboxylate transporter
MDMs: Monocyte-derived macrophages
MDSCs: Myeloid-derived suppressor cells
METTL: Methyltransferase-like protein
MM: Multiple myeloma
MMP: Matrix metalloproteinases
MMR: Mismatch repair
MLH1: MutL homolog 1
MOESIN: Membrane-organizing extension spike-associated protein
MoDCs: Monocyte-derived dendritic cells
MPC: Mitochondrial Pyruvate Carrier
MRE11: MRE11 homolog, double-strand break repair nuclease
MTHFD1L: Methylenetetrahydrofolate dehydrogenase (NADP^+^ dependent) 1-like
MUC20: Mucin 20
mTOR: Mechanistic target of rapamycin / mammalian target of rapamycin
MYB: Myb proto-oncogene, transcription factor
MYC/c-MYC: Cellular myelocytomatosis viral oncogene homolog
m6A: N6-methyladenosine
NADH/NAD^+^: Nicotinamide adenine dinucleotide
NF-κB: Nuclear factor-kappa B
NFAT: Nuclear factor of activated T-cells
NBS1: Nijmegen breakage syndrome 1
NCL: Nucleolin
NKR3: Natural Killer Cell Receptor 3
Nlrp3: NLR family pyrin domain containing 3
NMD: Nonsense-mediated mRNA decay
NMNAT1: Nicotinamide mononucleotide adenylyltransferase 1
Nrf2: Nuclear factor-erythroid 2-related factor 2
NSCLCs: Non-small cell lung cancers
NSUN2: NOP2/Sun RNA methyltransferase 2
NUSAP1: Nucleolar and spindle associated protein 1
OSCC: Oral squamous cell carcinoma
OXPHOS: Oxidative phosphorylation
p300: E1A binding protein p300
p53: Tumor protein p53
Pa: Palmitoylation
pCMBS: P-chloromercuribenzenesulfonic acid
PDAC: Pancreatic ductal adenocarcinoma
PDC: Pyruvate dehydrogenase complex
PDHA1: Pyruvate dehydrogenase E1-alpha subunit 1
PDHX: Pyruvate dehydrogenase complex component X
PDK1: Pyruvate dehydrogenase kinase 1
PD-L1: Programmed death-ligand 1
PDGFRB/PDGFRβ: Platelet-derived growth factor receptor beta
pDCs: Plasmacytoid dendritic cells
PFK: Phosphofructokinase
PFKFB4: 6-Phosphofructo-2-kinase
PHD: Pocket of the first PHD finger
PI3K: Phosphatidylinositol 3-kinase
PIs: Proteasome inhibitors
PK: Pyruvate kinase
PKM2: Pyruvate kinase M2
PML: Promyelocytic leukemia protein
POM121: Pore membrane protein 121
PROTAC: Proteolysis targeting chimera
PTBP1: Polypyrimidine tract-binding protein 1
PYCR1: Pyrroline-5-carboxylate reductase 1
PykF: Pyruvate kinase I
RARγ: Retinoic acid receptor gamma
RJA: Royal jelly acid
Rpo: RNA polymerase
ROS: Reactive oxygen species
RSV: Resveratrol
RUBCNL: Rubicon like autophagy enhancer
SHMT2: Serine hydroxymethyltransferase 2
SIRT/SIRT1: Silent information regulator 2 / sirtuin 1
SLFN5: Schlafen family member 5
SLC: Solute carrier
SMC4: Structural maintenance of chromosomes 4
SMAD: Mothers against decapentaplegic
SNAT2: Sodium-Coupled Neutral Amino Acid Transporter 2
SOX9: SRY-box transcription factor 9
SPOP: Speckle-type POZ protein
STAT3: Signal transducer and activator of transcription 3
TAMs: Tumor-associated macrophages
T-bet: T-box transcription factor expressed in T cells
TCA: Tricarboxylic acid cycle
TEAD1: TEA-domain transcription factor 1
TFEB: Transcription factor EB
TGF-β: Transforming growth factor-β
Th1 cell: Helper T cell 1
TIMs: Tumor-infiltrating myeloid cells
TIP60: Tat-interaction protein 60
TKIs: Tyrosine kinase inhibitors
TMZ: Temozolomide
TNBC: Triple-negative breast cancer
TNF-α: Tumor necrosis factor-α
TNFSF9: Tumor necrosis factor ligand superfamily member 9
TOP2A: DNA topoisomerase II alpha
TRAF6: Tumor necrosis factor receptor-associated factor 6
Tregs: Regulatory T cells
TRIM33: Tripartite motif-containing 33
TTK: Threonine tyrosine kinase
Ub: Ubiquitination
UK5099: 2-Cyano-3-(1-phenyl-1H-indol-3-yl)acrylic acid
USP39: Ubiquitin specific peptidase 39
VB124: 2-((5-(3-Cyclopropoxyphenyl)-1-phenyl-1H-pyrazol-3-yl)methoxy)-2-methylpropanoic acid
VCAM1: Vascular cell adhesion molecule 1
VEGF/VEGFR: Vascular endothelial growth factor / receptor
VHL: Von Hippel-Lindau
VAMP3: Vesicle-associated membrane protein 3
WDR74: WD repeat domain 74
Wnt: Wingless-related integration site
WWP2: WW domain-containing Protein 2
XBP1: X-box binding protein 1
XRCC: X-ray repair cross-complementing gene
YAP: Yes-associated protein
YBX1: Y-box binding protein 1
YEATS4: YEATS domain containing 4
YTHDF2: YTH domain-containing family protein 2
YY1: Yin yang 1
ZEB1: Zinc-finger E-box-binding homeobox 1
ZIP/ZIP1: Zinc influx transporter
